# A Versatile PDA(DOX) Nanoplatform for Chemo-Photothermal Synergistic Therapy against Breast Cancer and Attenuated Doxorubicin-Induced Cardiotoxicity

**DOI:** 10.1186/s12951-023-02072-1

**Published:** 2023-09-21

**Authors:** Siqi Geng, Qiang Feng, Chujie Wang, Ying Li, Jiaying Qin, Mingsheng Hou, Jiedong Zhou, Xiaoyu Pan, Fei Xu, Baoru Fang, Ke Wang, Zhangsen Yu

**Affiliations:** 1 School of Life and Environmental Sciences, Shaoxing University, Shaoxing, Zhejiang 312000 People’s Republic of China; 2 Laboratory of Nanomedicine, Medical Science Research Center, School of Medicine, Shaoxing University, Shaoxing, Zhejiang 312000 People’s Republic of China; 3 Department of Pathology, Shaoxing Hospital of Traditional Chinese Medicine, Shaoxing, Zhejiang 312000 People’s Republic of China; 4 Department of Ultrasound, Affiliated Hospital of Shaoxing University, Shaoxing, Zhejiang 312000 People’s Republic of China

**Keywords:** Polydopamine, Photothermal-chemotherapy synergistic therapy, Doxorubicin-induced cardiotoxicity, Oxidative stress

## Abstract

Photothermal therapy (PTT) is a highly clinical application promising cancer treatment strategy with safe, convenient surgical procedures and excellent therapeutic efficacy on superficial tumors. However, a single PTT is difficult to eliminate tumor cells completely, and tumor recurrence and metastasis are prone to occur in the later stage. Chemo-photothermal synergistic therapy can conquer the shortcomings by further killing residual tumor cells after PTT through systemic chemotherapy. Nevertheless, chemotherapy drugs' extreme toxicity is also a problematic issue to be solved, such as anthracycline-induced cardiotoxicity. Herein, we selected polydopamine nanoparticles (PDA) as the carrier of the chemotherapeutic drug doxorubicin (DOX) to construct a versatile PDA(DOX) nanoplatform for chemo-photothermal synergistic therapy against breast cancer and simultaneously attenuated DOX-induced cardiotoxicity (DIC). The excellent photothermal properties of PDA were used to achieve the thermal ablation of tumors. DOX carried out chemotherapy to kill residual and occult distant tumors. Furthermore, the PDA(DOX) nanoparticles significantly alleviate DIC, which benefits from PDA's excellent antioxidant enzyme activity. The experimental data of the chemotherapy groups showed that the results of the PDA(DOX) group were much better than the DOX group. This study not only effectively inhibits cancer but tactfully attenuates DIC, bringing a new perspective into synergistic therapy against breast cancer.

**Graphical Abstract:**

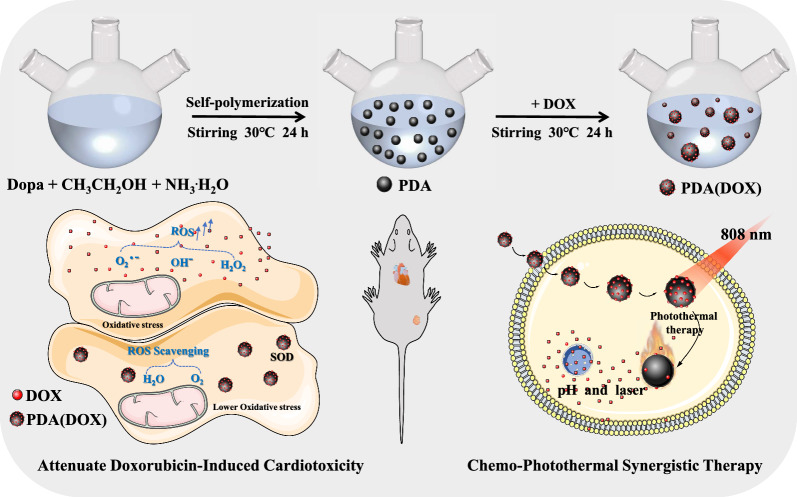

**Supplementary Information:**

The online version contains supplementary material available at 10.1186/s12951-023-02072-1.

## Introduction

Breast cancer has become one of the most common malignant tumors in women. According to GLOBOCAN 2020 [1], female breast cancer has surpassed lung cancer to become cancer with the highest incidence rate globally. The incidence rate of breast cancer ranks first in 159 out of 185 countries worldwide, with a morbidity and mortality rate of 47.8/100,000 and 13.6/100,000, respectively, ranking first among women’s cancer. Therefore, it can be seen that the situation of prevention, diagnosis, and treatment of breast cancer is dire. In the current clinical treatment of breast cancer, surgery combined with chemotherapy or radiotherapy is the primary treatment method. However, these treatment technologies have many defects, such as obvious trauma, drug toxicity, side effects, and patient pain [2, 3]. Therefore, developing efficient and safe therapeutic drugs is crucial for achieving excellent treatment efficacy.

With the rapid development of nanomedicine, using advanced nanoparticles to design versatile nano drugs for tumor diagnosis and treatment has gradually become a research hotspot in oncology. Among them, PTT uses near-infrared light to irradiate the photothermal agents enriched in the tumor, convert light energy into heat energy, and generate local high temperatures to kill tumor cells. Because PTT is precise and controllable, non-invasive, and has promising therapeutic efficacy, low systemic toxicity, and other advantages, it has become a tumor treatment technology with broad application prospects [4–7]. Moreover, the photothermal properties of photothermal agents are crucial in PTT. Nanogold, black phosphorus, Prussian blue, polypyrrole, and other nano-photothermal agents synthesized based on nanotechnology are emerging endlessly, providing nano-pharmaceuticals with exceedingly good photothermal performance and biosafety for the implementation of tumor PTT. These kinds of photothermal pharmaceutical preparations have achieved excellent PTT efficacy at the level of tumor cells in vitro and tumor-bearing mice in vivo [8–12]. However, because the temperature distribution constructed by PTT is challenging to measure accurately in real time, the boundary of the temperature field is difficult to control. When using PTT mode alone for treatment, the usual situation is that the heated tumor site can be quickly cured. However, residual tumor cells will still be accessible to relapse and metastasize in the boundary area of PTT. Therefore, using PTT alone to treat tumors remains a significant technical challenge in accurately determining the photothermal heating boundary and achieving safe and effective therapeutic effects. In recent years, chemo-photothermal synergistic therapy has become a more effective treatment strategy [13, 14]. PTT is used to carry out precise thermal ablation of the tumor and further use of chemotherapy drugs to continuously kill residual tumor cells to inhibit tumor recurrence and metastasis from achieving an excellent therapeutic effect [15–17]. The commonly used chemotherapeutic drugs in chemo-photothermal synergistic therapy mainly include doxorubicin [18], 7-ethyl-10-hydroxycamptothecin [19], and cisplatin [15, 20].

Recently research has shown that synergistic treatment for tumors has a more significant effect than the single treatment mode. Nevertheless, the shortcoming of chemotherapy-based synergistic treatment technologies with serious toxic side effects on normal tissues, such as adriamycin-induced cardiomyopathy, urgently needs to be addressed. The incidence rate of doxorubicin (DOX) induced cardiomyopathy depends on the cumulative amount of DOX. When the cumulative amount of DOX exceeds 700 mg/m^2^, the incidence rate increases to 18%–48%. Even when the cumulative dose of DOX exceeds 550 mg/m^2^, 26% of patients will suffer from heart failure. Unfortunately, this cardiomyopathy is progressive, irreversible and can still appear after several years of terminating chemotherapy [21]. And as a consequence, while using nanotechnology to build synergistic therapeutic nano agents based on chemotherapy drugs to improve efficacy, it is an urgent problem to alleviate the side effects of chemotherapy drugs on the heart. DOX-induced myocardial injury results from multiple factors, such as the production of reactive oxygen species, mitochondrial damage, calcium overload, and apoptosis, among which oxidative stress plays a vital role in the toxic effects of DOX [22, 23]. Numerous studies have suggested that using drugs with antioxidant activity to reduce oxidative stress levels in myocardial cells can effectively alleviate DIC [24, 25]. In recent years, a new type of artificial enzyme, nanozyme, has multiple excellent enzyme activities, such as superoxide dismutase, catalase, and peroxidase. Based on these enzyme activities, antioxidant and anti-inflammatory effects can be achieved, significantly relieving oxidative stress disease. Common nanozymes include ferric oxide [26], cerium oxide [27], Prussian blue [28], and polydopamine [29]. Based on different types of nanozymes, many excellent research results have been achieved in neurodegenerative diseases [30, 31], organ ischemia/reperfusion injury [32, 33], drug-induced acute organ injury [24, 34, 35], chronic inflammatory diseases therapeutics [36], and detection of small molecules in vitro.

Polydopamine nanoparticles (PDA) are nanoscale melanin particles with excellent properties, such as stable physical and chemical properties, good dispersion, biocompatibility, and biodegradability [37, 38]. Furthermore, PDA has strong absorption and high photothermal conversion efficiency in the near-infrared light band. A recent investigation demonstrated that melanin-like PDA could effectively convert NIR light into heat and kill cancer cells in vitro and in vivo. PDA is a potential photothermal agent for superficial tumor treatment [39, 40]. Meanwhile, the phenolic hydroxyl groups on the surface of PDA have excellent antioxidant performance and easy-to-achieve surface modification, such as loading metal ions, small molecule drugs, *etc.*, to enhance its function to achieve multimodal synergistic treatment of tumors [5, 41, 42].

**Scheme 1:**
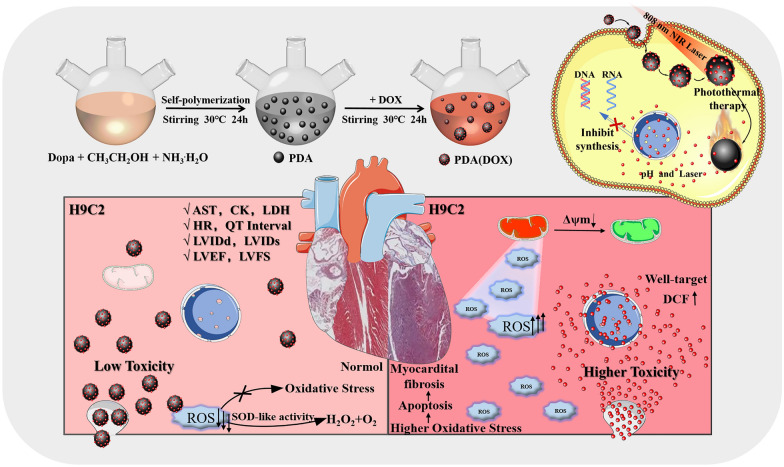
Schematic illustration of PDA(DOX) nanoplatform for chemo-photothermal synergistic therapy against breast cancer and attenuated doxorubicin-induced cardiotoxicity

In this work, we synthesized a novel PDA(DOX) nanoplatform with highly effective chemo-photothermal synergistic treatment efficacy and alleviated the toxic and side effects of chemotherapy drugs (Scheme 1). PDA(DOX) nanoparticles could be achieved by the one-pot method of PDA and DOX in a mild solution. The abundance of aromatic rings on the PDA surface makes it possible to load chemotherapy drugs via π–π stacking and/or hydrogen binding and plays an antioxidant role [43, 44]. Additionally, we revealed the influence of particle size of PDA nanoparticles on their photothermal properties and enzyme activity. In addition, the treatment results of the 4T1 tumor-bearing mouse model and tumor cells in vitro showed that the chemo-photothermal synergistic therapy based on PDA(DOX) nanoparticles had a significant effect. Moreover, the efficacy of PDA(DOX) on myocardial injury due to chemotherapy in tumor-bearing mice was further evaluated. We hope that by combining PDA and DOX on a single platform, our work will achieve synergistic therapy and attenuate the myocardial toxicity induced by the chemotherapy drug DOX, which is expected to provide an effective and safe solution for breast cancer to heal.

## Results and discussion

### Preparation and characterization of PDA(DOX) nanoplatform

**Fig. 1:**
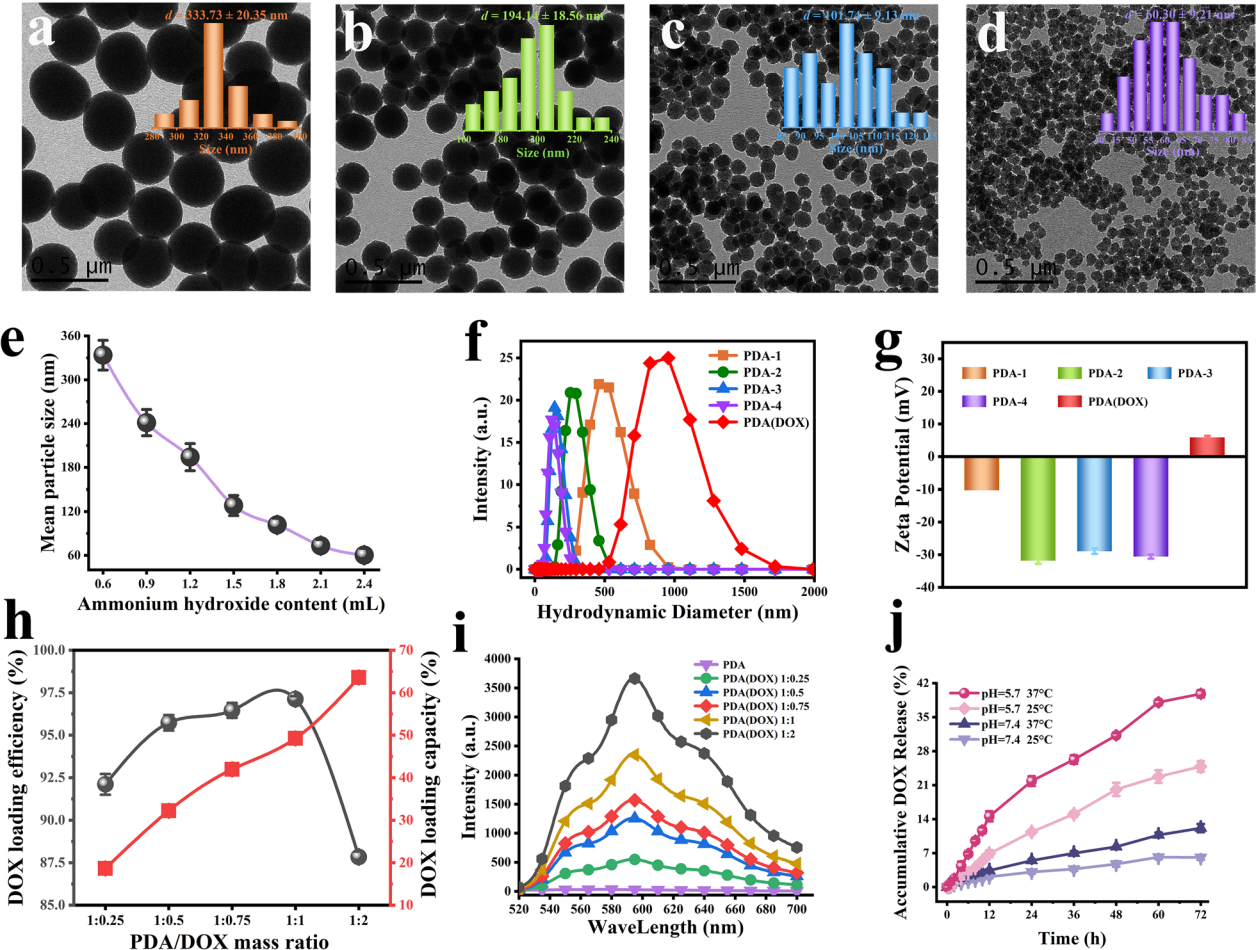
Synthesis and characterization of PDA-*i* and PDA(DOX) nanoparticles. **a**–**d** TEM images and size distributions of PDA-*i*; **e** Relationship between mean particle size and ammonium hydroxide content; **f**, **g** DLS and zeta potential of PDA-*i* and PDA(DOX); **h** The DOX loading efficiency and capacity of PDA(DOX) nanoparticles obtained under different PDA/DOX mass ratio reaction conditions; **i** Fluorescence spectrum of PDA-*i* and PDA(DOX) of various DOX loading capacity; **j** Release kinetics curves of PDA(DOX) under different pH or temperature

Transmission electron microscopy images (Fig. 1a–d and Additional file 1: Figure S1a–c) showed PDA nanoparticles with different sizes. The obtained PDA nanoparticles exhibited a spherical shape with average diameters and narrow size distributions of 333.73 ± 20.35 nm, 194.14 ± 18.56 nm, 101.74 ± 9.13 nm, and 60.30 ± 9.21 nm [named PDA-*i* (*i* = 1–4)]. Furthermore, the size of PDA nanoparticles decreases with the increase in ammonia concentration (Fig. 1e), which further confirms that the ammonia concentration in the reaction solution could adjust the size of PDA nanoparticles. And it was considered that the nanoparticles with small size have the advantages of large specific surface area, more active sites for binding small molecular drugs on the surface, and easy-to-achieve intravenous administration and excretion through a metabolic pathway. Therefore, PDA-4 was chosen as a drug carrier loaded with DOX under mildly magnetic stirring at 30 °C for 24 h. The average hydrodynamic diameter of PDA-4 and PDA(DOX) was about 120 nm and 955 nm (Fig. 1f), and the Zeta potential changed from a negative value of − 30.55 ± 0.64 mV to a positive value of 5.91 ± 0.42 mV (Fig. 1g), respectively. Meanwhile, compared with PDA, the PDA(DOX) emerged with the characteristic absorption peak of DOX at 488 nm (Additional file 1: Figure S2b). The characterization results from TEM, DLS, zeta potential, and UV–vis-NIR confirmed the successful regulation of PDA nanoparticle size and PDA(DOX) nanoplatform preparation. The mass concentrations of PDA-*i*, PDA(DOX) nanoparticles, and DOX were determined by the standard curves of their mass concentrations vs. their corresponding absorbance values at the wavelength (808 nm, 488 nm) (Additional file 1: Figure S3).

Figure 1h showed that the highest DOX loading efficiency of PDA(DOX) obtained under the different PDA/DOX mass ratio conditions is 97.13 ± 0.16%, and the loading capacity increased continuously from 18.65 ± 0.14% to 63.58 ± 0.27%. In addition, the changing trend of fluorescence intensity of PDA(DOX) excited by 488 nm laser is entirely consistent with the rising trend of DOX loading capacity in the samples (Fig. 1i). Therefore, comprehensive consideration of the DOX loading capacity and dispersion of PDA(DOX), the mass ratio of 1:0.75 was selected to synthesize PDA(DOX) for subsequent experiments. Furthermore, we further assessed the accumulative DOX release characteristics of PDA(DOX) under different pH and temperature conditions. As shown in Fig. 1j, the accumulative release of DOX is 39.82% in PBS (pH 5.7, tumor acidic microenvironment) after 72 h. In particular, the DOX release is only 6.12% in the pH 7.4 PBS (normal tissue microenvironment). In addition, the release rate of DOX increased significantly as the buffer temperature increased. These results observations indicated that using PDA(DOX) for tumor chemotherapy is expected to reduce the toxicity of DOX to normal tissues. Meanwhile, the increase in tumor tissue temperature caused by PTT helps improve the release rate of DOX.

### Photothermal and antioxidation performance evaluation

To investigate the photothermal performance of PDA with various sizes and PDA(DOX) nanoparticles, the absorption spectra, temperature rise curves, temperature rising and falling curves, and repeatability curves were detected. The absorption spectra showed that PDA-*i* and PDA(DOX) have excellent absorption performance in the visible and near-infrared regions, which gradually weakened as the nanoparticle size decreased. In addition, the absorption performance of PDA(DOX) obtained by PDA-4 loaded with DOX is significantly enhanced, which is very beneficial to photothermal therapy (Additional file 1: Figure S2a). Simultaneously, the characteristic absorption peak of PDA(DOX) appeared at 400–560 nm, indicating that DOX was successfully loaded on the surface of PDA, and the color difference between PDA and PDA(DOX) dispersion is consistent with the absorption spectrum change (inserted picture in Additional file 1: Figure S2b).

**Fig. 2:**
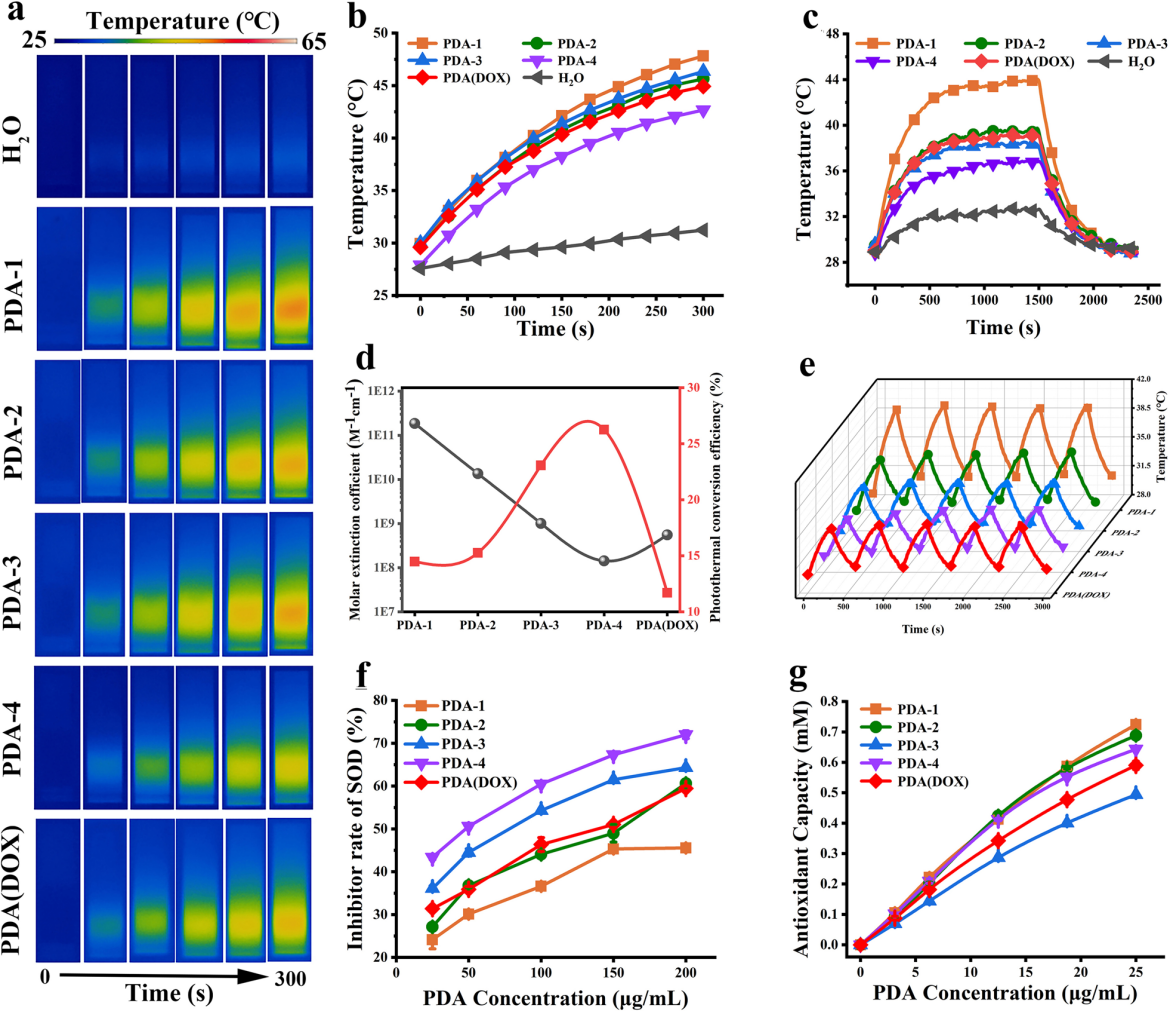
Photothermal and antioxidation performance of PDA-*i* and PDA(DOX) nanoparticles. **a** Thermal images recorded for PDA-*i* and PDA(DOX); **b** Photothermal temperature rise curves (100 μg/mL); **c** Temperature rise and fall curves (50 μg/mL); **d** Photothermal conversion efficiencies and Molar extinction coefficients; **e** Photothermal repeatability curves under five on/off cycles (50 μg/mL); **f** Inhibitor rate of SOD; **g** Antioxidant Capacity of PDA-*i* and PDA(DOX)

Figure 2b and Additional file 1: Figure S4 showed that the temperature of PDA-*i* and PDA(DOX) dispersions raised quickly with the increasing nanoparticle mass concentration compared with the deionized water group. And the temperature rises continuously with the extension of irradiation time, which is entirely consistent with the temperature change trend shown in the thermographic images (Fig. 2a). Note that the increased temperature (Δ*T*) of PDA-1 (100 μg/mL) could reach 17.88 °C under 1.5 W laser irradiation. Under the same condition, the Δ*T* was 15.32 °C of the PDA(DOX) dispersion with 100 μg/mL, which was enough to result in necrosis and apoptosis of tumor cells.

Moreover, the photothermal conversion efficiency (η) of PDA-*i* and PDA(DOX) were measured and calculated by temperature rising and falling curves. PDA-*i* and PDA(DOX) (50 μg/mL) could increase to 43.80, 39.47, 38.16, 36.62, and 39.08 °C after approximately 20 min laser irradiation. However, deionized water only reached 32.60 °C (Fig. 2c). The η of PDA-*i* and PDA(DOX) went to 13.68%, 13.77%, 20.51%, 22.65%, and 10.60% (Fig. 2d), calculated by Additional file 1: Equation S1. And the temperature falling curves were used to calculate the τ_s_ and parameter *h*S (Additional file 1: Figure S5). The photothermal conversion efficiencies and the specific data used in the calculation process were listed in Additional file 1: Table S1. Furthermore, Fig. 2e showed that the photothermal stability was assessed via the photothermal repeatability curves. PDA-*i* and PDA(DOX) were not significantly changed during the 5 cycles, and the maximum temperatures stabled at about 40.69, 37.26, 36.20, 35.12, and 36.22 °C, respectively. Therefore, it was suggested that PDA-*i* and PDA(DOX) had outstanding photothermal stability.

The absorption performance of photothermal agents on 808 nm wavelength lasers directly affects their photothermal properties. Thus, the differences in the absorption property of PDA-*i* and PDA(DOX) nanoparticles with different particle sizes were further analyzed through theoretical calculations. We calculated the absorption cross-section, absorption efficiency factor, and molar absorption coefficient of PDA nanoparticles using particle size, absorbance value at 808 nm, and concentration of PDA nanoparticles. As shown in Additional file 1: Table S2, the absorption cross-section (σ_808 nm_) and absorption efficiency factor (Q_808 nm_) values of PDA-1 were almost 1300 and 40 times as much as that of PDA-4, respectively (Additional file 1: Equations S2, S3). Moreover, consistent with the σ_808 nm_, the molar absorption coefficient (ε_808 nm_) is also proportional to the third power of the particle size (Additional file 1: Equations S4). Therefore, with the decrease of PDA-*i* size, the ε_808 nm_ of nanoparticles also decreased significantly. For example, as shown in Fig. 2d and Additional file 1: Table S2, the ε_808 nm_ of PDA-1 is 1.86 × 10^11^ M^−1^ cm^−1^, while that of PDA-4 is only 1.43 × 10^8^ M^−1^ cm^−1^.

The phenolic hydroxyl groups on the surface of PDA nanoparticles are easy to lose electrons and be oxidized. Therefore, it can be an antioxidant agent to reduce oxidative stress and protect cells from toxic damage caused by excessive active oxygen. To verify this, we evaluated the ability of PDA-*i* and PDA(DOX) to scavenge ROS in vitro by SOD activity and total antioxidant capacity. As displayed in Fig. 2f, the SOD enzyme activity continued strengthening due to increasing PDA concentration, indicating that the PDA-*i* is an excellent antioxidant. Additionally, with the decrease in PDA size, the activity of the SOD enzyme also showed a significant increasing trend. As we know, the SOD enzyme activity of PDA nanoparticles is the property of the phenolic hydroxyl group on its surface. And with the decrease in the particle size of PDA nanoparticles, its specific surface area increased significantly, and the proportion of phenolic hydroxyl groups on the surface increased, thus enhancing the activity of the SOD enzyme. Such as, when the concentration is 200 μg/mL, PDA-4 has the highest activity, and the inhibition rate of superoxide is up to 72.00 ± 0.90%. However, the surface of PDA-4 loaded with DOX consumed some phenolic hydroxyl, which reduced its SOD enzyme activity. At the same concentration, the inhibition rate was 59.46 ± 0.58%. Furthermore, ABTS was used to evaluate the free radical scavenging of PDA-*i* and PDA(DOX). Figure 2g suggests the antioxidant capacity of PDA(DOX) reached 0.59 ± 0.01 mM at a concentration of 25 μg/mL. According to the results above, PDA(DOX) is an efficient antioxidant for scavenging ROS in vitro.

Based on all the discussions above, we could conclude that the particle size of PDA nanoparticles decreases, resulting in a decrease in their absorption cross-section and molar absorption coefficient. Although the photothermal conversion efficiency increases with the reduction of nanoparticles size, the overall temperature rise effect is still weakened. In addition, the enzyme activity sites increased on the surface of PDA with a small size, which enhances the enzymatic activity of nanoparticles and significantly improves the ability to scavenge free radicals.

### Cytotoxicity and in vitro curative effects

**Fig. 3:**
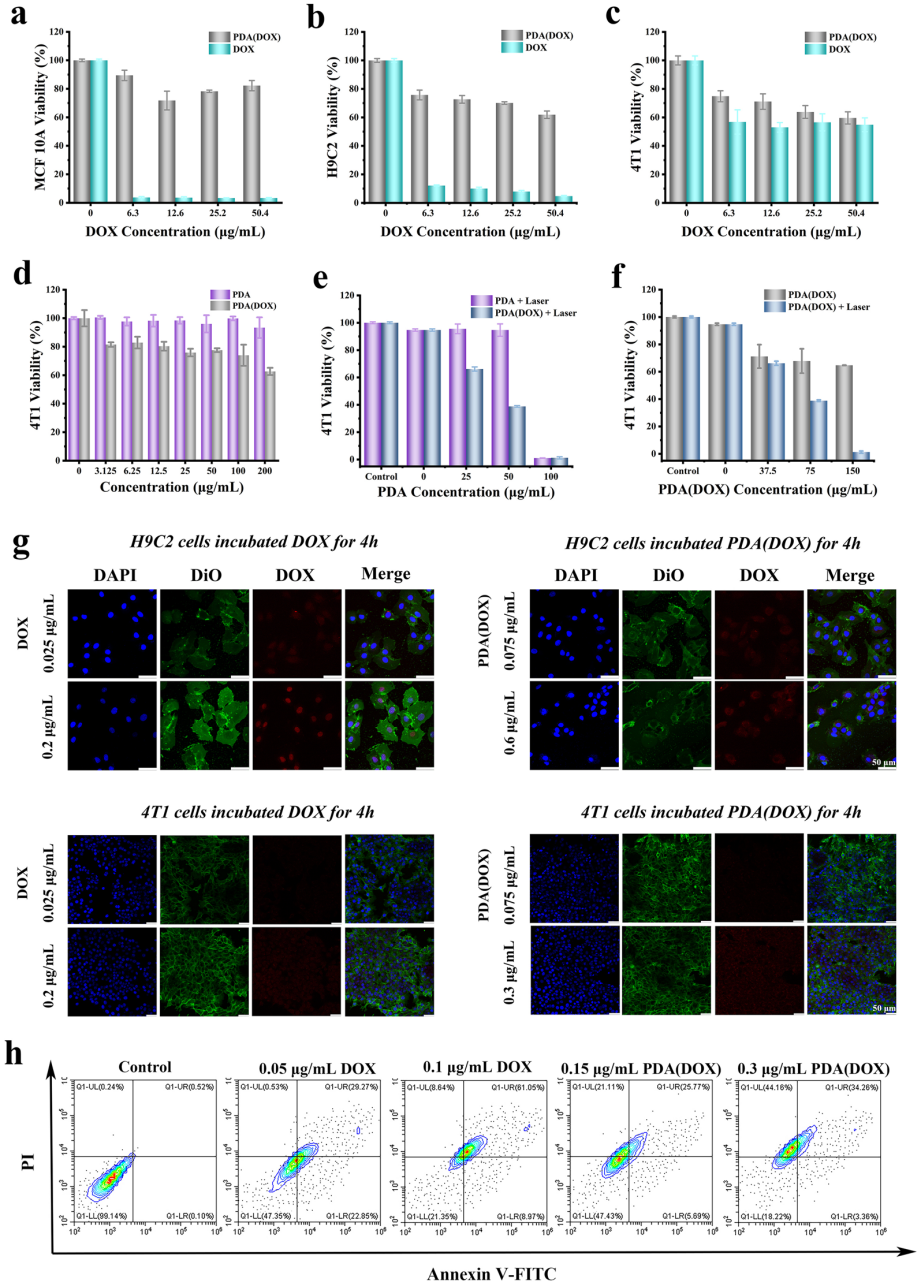
Cytotoxicity and in vitro curative effects. **a**–**c** The cytotoxicity of various concentrations of PDA(DOX) and DOX on MCF-10A cells, H9C2 cells, and 4T1 cells; **d** The cytotoxicity of various concentrations of PDA and PDA(DOX) on 4T1 cells; **e** The photothermal killing effects of PDA or PDA(DOX) on 4T1 cells after NIR irradiation at a laser power of 1.5 W for 5 min; **f** The synergetic effect of photothermal therapy and chemotherapy on 4T1 cells; **g** Confocal microscope images of H9C2 and 4T1 cells uptake DOX and PDA(DOX) for 4 h with different concentrations; **h** Flow cytometric analysis of apoptosis in 4T1 cells after incubated with DOX or PDA(DOX) for 24 h and Annexin V-FITC/PI double staining

PDA(DOX) cytotoxicity was quantitatively evaluated on cancer and normal cells by the MTT method. Figure 3a, b, and Additional file 1: Figure S6 showed that PDA(DOX) with lower toxicity to MCF-10A human mammary epithelial and H9C2 rat cardiomyocyte cells compared to the same concentration of DOX. Surprisingly, when the concentration of DOX is 6.30 μg/mL, the viability of MCF-10A and H9C2 cells in the PDA(DOX) group is 89.44 ± 3.64% and 75.75 ± 3.45%, which is almost 24 and 6 fold that in the DOX group, respectively. In the meantime, after PDA(DOX) or DOX were co-incubated with 4T1 cells, the results in Fig. 3c showed that when the DOX concentration reached 50.4 μg/mL, 4T1 cells viability of the PDA(DOX) and DOX groups are 59.63 ± 4.30% and 54.85 ± 4.84%, respectively. However, after co-incubation with 200 μg/mL PDA nanoparticles for 24 h, the viability of 4T1 cells was above 93.38 ± 7.28% (Fig. 3d). What’s more, flow cytometry results showed that DOX and PDA(DOX) increased the apoptosis rates of chemotherapy-induced 4T1 cells (Fig. 3h). The above results illustrated that the PDA(DOX) nanoplatform could significantly alleviate the toxicity of DOX to normal cells without weakening the chemotherapy-killing effect of DOX on 4T1 cells. The phagocytic experiment results showed that the phagocytic capacity of H9C2 cells on DOX and PDA(DOX) was increased with the prolongation of coincubation time (Fig. 3g, Additional file 1: Figures S7, S8). Additionally, DOX has a perfect nuclear-targeting effect on H9C2 cells, while PDA(DOX) as a carrier weakens DOX’s nuclear-targeting effect on H9C2 cells, thereby alleviating DOX’s toxicity to H9C2 cells. On the other hand, the distribution of DOX and PDA(DOX) phagocytosed by 4T1 cells lacks apparent nuclear targeting and is evenly distributed within the cytoplasm. Thus, the killing rates of these two drugs on 4T1 cells are the same. Moreover, compared to 2 h, 4T1 cells showed a decrease in phagocytosis of both drugs at 4 h, indicating that DOX was effluxed by 4T1 cells.

We further investigated the therapeutic effects of PDA(DOX) nanoparticles on 4T1 cells by chemo-photothermal synergistic therapy. Fig. 3e and f showed that the viability of 4T1 cells treated with PDA(DOX) under 808 nm laser irradiation decreases significantly as the concentration increase. When the PDA(DOX) was 75 μg/mL, the corresponding cell mortality rate was 5.30% in the PTT group with the same PDA concentration (50 μg/mL) and 32.13% in the chemotherapy group with the same DOX concentration. However, surprisingly, the cell mortality rate of the PDA(DOX) + laser group reached 61.15%. The survival rate of 4T1 cells approached about 0% after the implementation of chemo-photothermal synergistic therapy when the PDA(DOX) concentration was 150 μg/mL. The in vitro cell therapy experiment results demonstrated that the combination therapy mode based on PDA(DOX) achieves excellent synergistic treatment efficacy compared with PTT and chemotherapy alone.

### In vivo curative effects

**Fig. 4:**
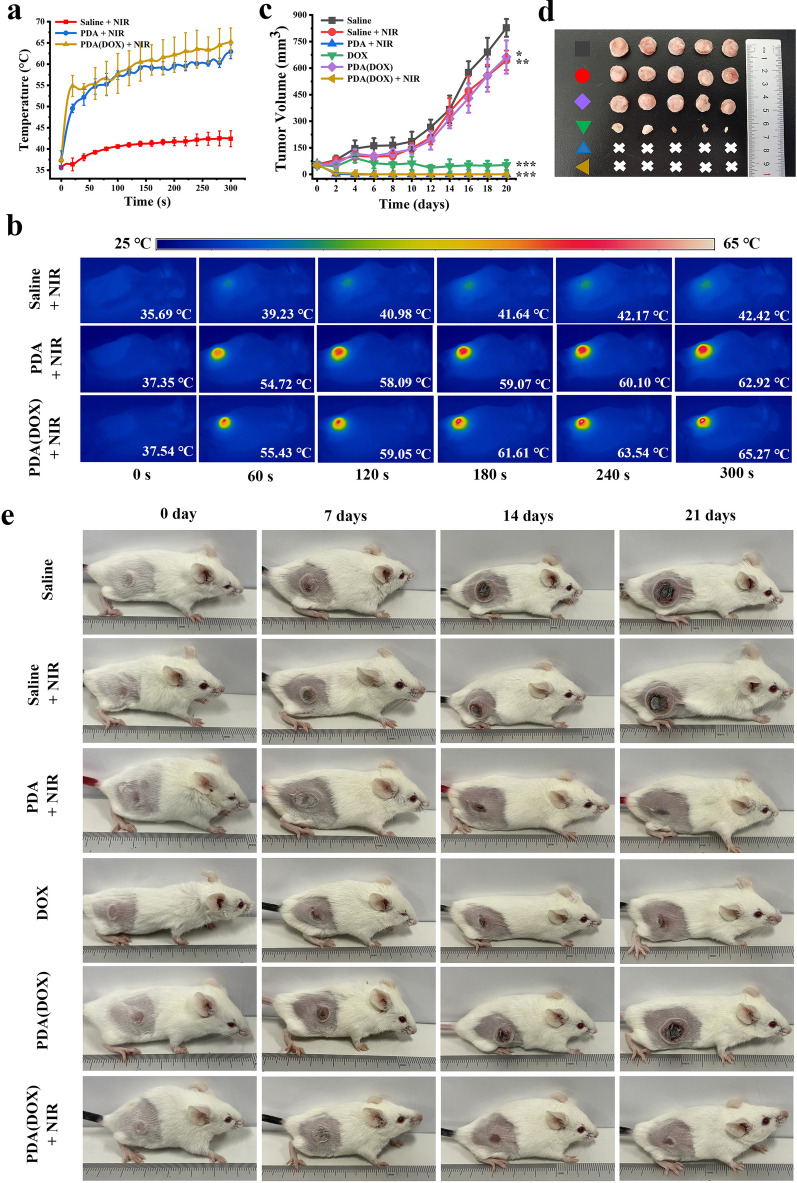
In vivo synergetic chemo-photothermal therapy of tumor. **a**, **b** Temperature rise curves and Thermal images of 4T1 tumor-bearing mice irradiated by 808 nm laser at a laser power of 2 W/cm^2^ for 5 min; **c** Tumor growth curve within 21 days after different treatments; **d** Photographs of the excised tumors of mice after sacrifice; **e** 4T1 tumor-bearing mice images on the 0, 7, 14, and 21 days after different treatments

To shed light on the in vivo curative potential of PDA(DOX), 4T1 tumor-bearing Balb/c mice were prepared for the following experiments. Figure 4a showed the temperature of the tumor surface on the PDA(DOX) + NIR group could rise to 65.27 ± 3.25 °C, while the saline + NIR group only increased to 42.42 ± 1.87 °C. After injection in the tumor with PDA(DOX) or PDA, the tumor showed a rapid temperature rise above 54 °C by thermogram pictures under the 808 nm laser radiation (2 W /cm^2^) for 1 min (Fig. 4b). Figure 4c showed that the tumor volumes of the PDA(DOX) group were decreased compared with the saline group. However, the tumor growth curve was still an upward trend, mainly because the therapeutic effect of just one intratumor injection of chemotherapy drugs is limited. In addition, the tumor inhibition rates of DOX, PDA + NIR, and PDA(DOX) + NIR groups were calculated from the tumor growth curves to be 93.59%, 100%, and 100%, respectively. Each group's tumor photos and weight of mice 21 days after treatment were consistent with the above results (Fig. 4d and Additional file 1: Figure S9a). The digital pictures in different treatment groups display tumor volume changes after chemo-photothermal synergistic therapy (Fig. 4e). What’s more, the weight changes of mice and the results of H&E staining tissue sections of their main organs showed that neither the synergistic therapy nor chemotherapy had toxicity to mice after 21 days of synergistic treatment (Additional file 1: Figures S9b, S10). In summary, PDA(DOX) could effectively kill tumor cells through synergistic chemo-photothermal therapy and achieve tumor ablation. Therefore, it is expected to become a valuable and safe drug for breast cancer treatment.

**Fig. 5:**
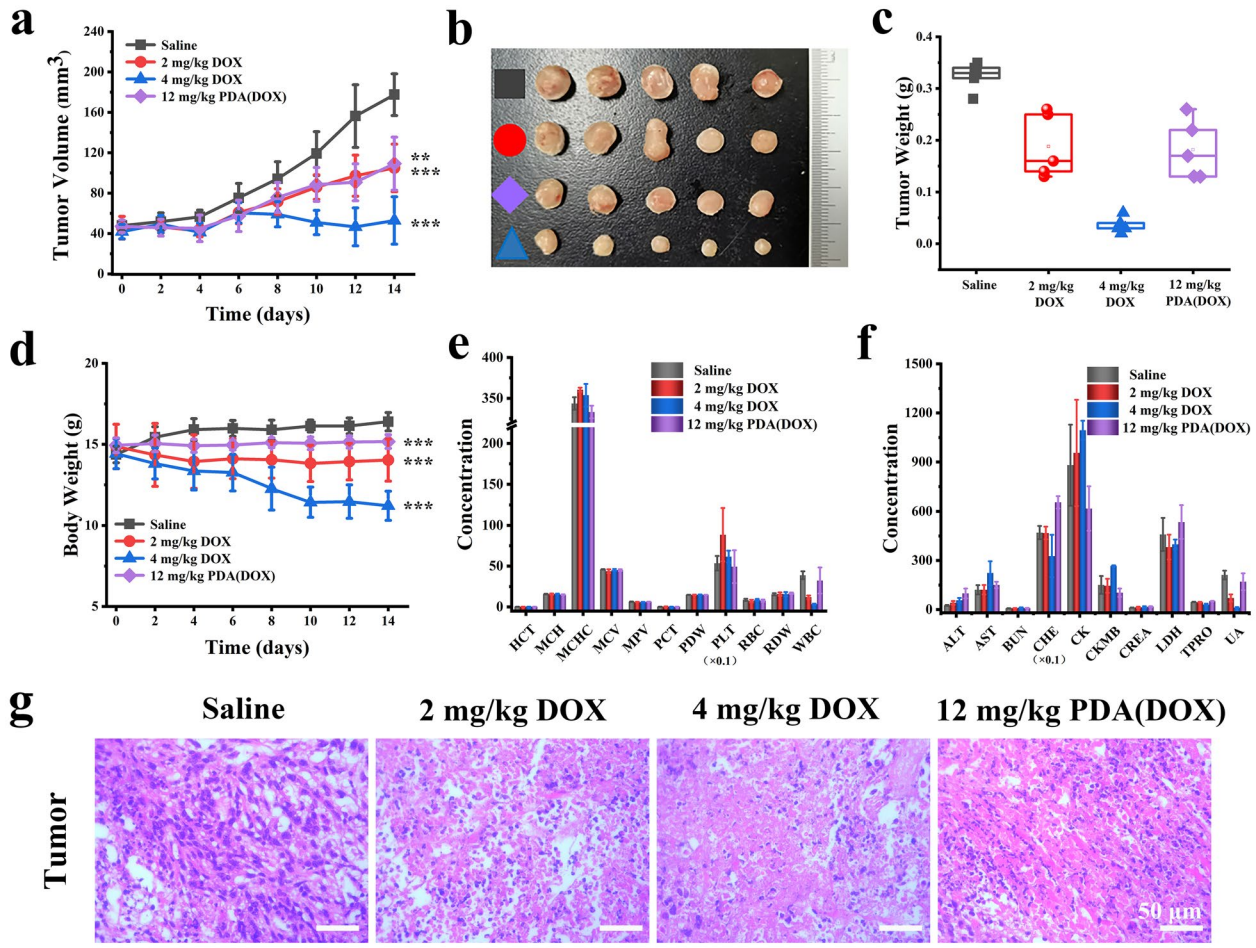
In vivo anticancer efficiency of PDA(DOX) by chemotherapy. **a** Tumor growth curves within 14 days after different drug treatments; **b**, **c** Photographs and weight of the excised tumors of mice after sacrifice; **d**–**f** Body weight, blood routine test, and serum biochemical indices of chemotherapy mice after 14 days. **g** H&E staining images of mice tumors after different chemotherapy treatments for 14 days

Inspired by the hopeful tumor-bearing mice synergistic therapy in vitro results, we further studied the curative effects of PDA(DOX) on chemotherapy in vivo. Figure 5a showed that after 14 days of chemotherapy, the tumor growth inhibition rate of the PDA(DOX) group with an intravenous injection dose of 12 mg/kg could reach 38.51%, which was consistent with the efficacy of the DOX group of 2 mg/kg. However, the tumor suppression rate of the 4 mg/kg group was as high as 70.20%, significantly higher than that of the other two experimental groups. The photos and weights of the ex vivo tumor and 4T1 tumor-bearing mice images on the 0, 7, and 14 days after different treatment results are consistent with the tumor growth curves (Fig. 5b, c, Additional file 1: Figure S11a). In contrast with the control group, the pathological consequences of H&E staining in the tumor tissues of DOX and PDA(DOX) groups all showed prominent necrosis and apoptosis (Fig. 5g). To demonstrate the effect of chemotherapy more clearly, the NIR laser power density was reduced to 0.8 W /cm^2^ in photothermal therapy, thereby preventing tumor cells from being completely killed after PTT. Subsequently, chemotherapy was implemented to further inhibit tumor growth. As shown in Additional file 1: Figure S13, the fluorescence imaging results of PDA(DOX)@ICG at different time points after intravenous injection indicated that the drug could enter tumor tissue and exert chemotherapy effects. After 0.8 W/cm^2^ laser power irradiation for 5 min, the temperature of the tumor site in the PDA(DOX) i.m. + NIR + i.v. group increased by about 7.85 °C. Based on the better photothermal effect of intratumoral injection of PDA(DOX), when the two groups injected the same dose of PDA(DOX) within 14 days, PDA(DOX) i.m. + NIR + i.v. showed a better combination treatment effect by the tumor volume (Additional file 1: Figures S13d, e, S14a–e).

While DOX achieves antitumor efficacy, its toxicity cannot be ignored. A high concentration of DOX has an excellent therapeutic effect but also shows extreme toxicity. Figure 5d showed that the weight loss rate of mice in the experimental group with 2 mg/kg and 4 mg/kg DOX treatment doses is 5.61%, and 22.18%, respectively. Fig. 5e and f showed the blood routine, and serum biochemical indicators test results of each experimental group of mice. The white blood cell levels in the DOX groups were significantly reduced, and the AST indicators increased. The hematology and biochemical test results of the PDA(DOX) group were consistent with those of the control group. In addition, the organ photos of the chemotherapy groups mice showed that DOX chemotherapy caused very severe spleen atrophy in the mice (Additional file 1: Figure S11b). As Additional file 1: Figure S12 showed, the organ coefficients of the spleen in the 2 mg/kg and 4 mg/kg DOX chemotherapy groups of mice decreased by 46.7% and 86.1%, respectively. According to literature reports, spleen atrophy is mainly caused by DOX damage to the CD169+ macrophage population of peripheral immune cells in the spleen [45]. The PDA(DOX) group obtained the opposite result, successfully reversing severe splenic atrophy induced by DOX. However, mice treated with PDA(DOX) chemotherapy showed slight enlargement of the spleen and liver, possibly due to PDA(DOX) being engulfed more by these two organs and leading to temporary congestive organ tumefaction. The above experimental results indicate that loading DOX with PDA significantly reduces the toxicity of DOX.

### In vitro and in vivo mitigation of DOX-induced myocardial toxicity effective

**Fig. 6:**
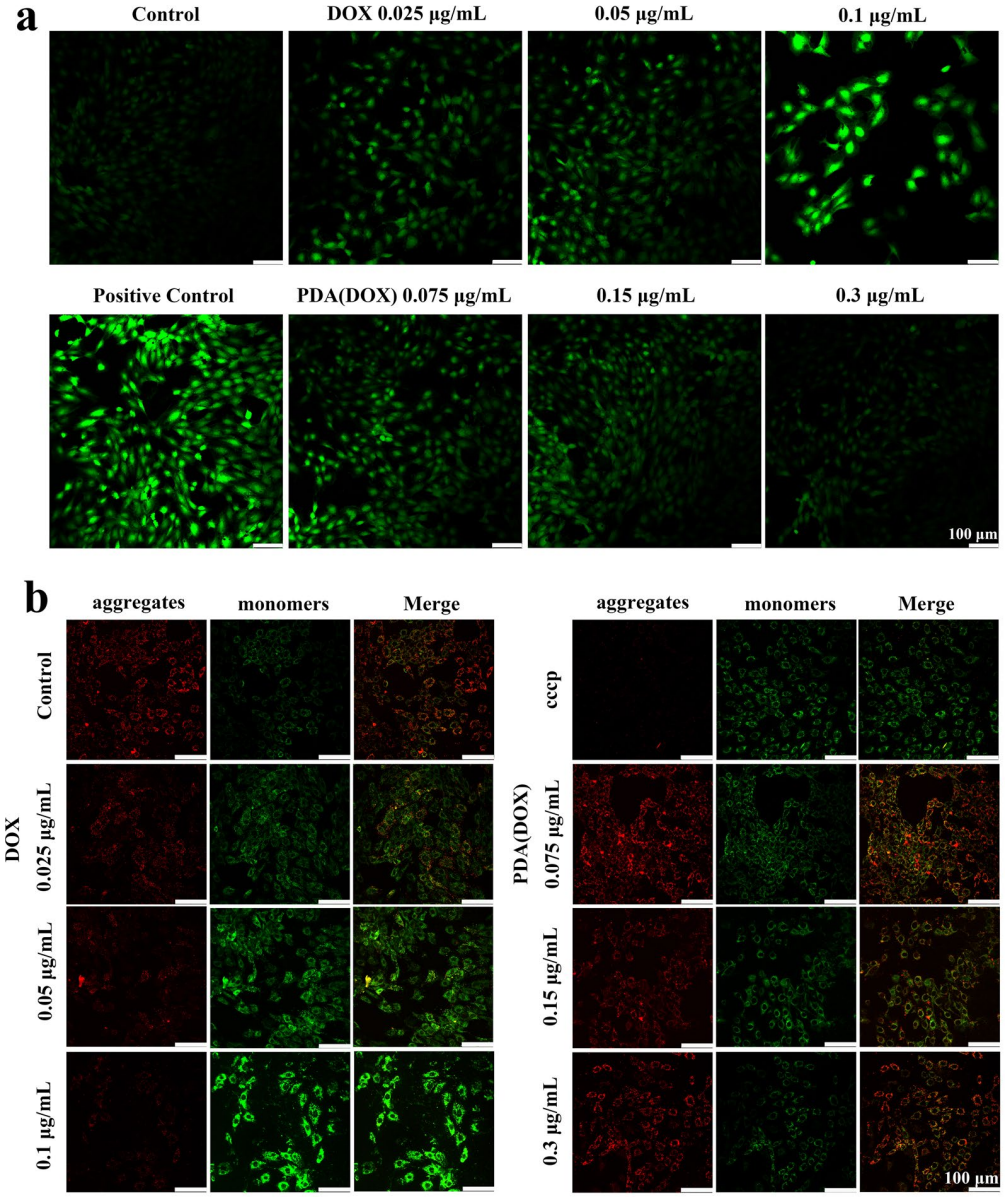
In vitro mitigation of DOX-induced myocardial toxicity effective. **a** DCF fluorescence images for intracellular detection of ROS production on H9C2 cells after incubated with DOX or PDA(DOX) for 24 h; **b** JC-1 fluorescence images for cell mitochondrial membrane potential detection on H9C2 cells after incubated with DOX or PDA(DOX) for 24 h

We first investigated the ROS scavenging activity of PDA(DOX) by DCFH-DA. Figure 6a shows that fluorescence intensity increases gradually with increasing DOX concentration. When the DOX concentration was 0.1 μg/mL, DCFH-DA emitted intensive green fluorescence, revealing a high level of ROS. In contrast, the same DOX concentration of PDA(DOX) has detected weak fluorescence. The above data indicated that DOX could induce oxidative stress imbalance in H9C2 cells, producing a large amount of oxygen-free radicals and further causing severe myocardial damage. The chemotherapy drug carrier PDA plays an essential role in alleviating myocardial cell injury.

Mitochondrial membrane potential is an essential indicator of cellular health and functional status [46]. And the changes in mitochondrial membrane potential were judged by changes in the intensity of red fluorescence of JC-1 aggregates and green fluorescence of JC-1 monomers. Figure 6b clearly shows that the red fluorescence intensity gradually decreased with the increase of DOX. In contrast, the green fluorescence intensity significantly increased, exceeding the green fluorescence intensity of the positive control group. The change from red to green fluorescence indicated that JC-1 did not aggregate in the mitochondrial matrix and changed from polymer to monomer, which proved that the cells in the early stage of apoptosis and the membrane potential decreased. On the contrary, the PDA(DOX) group with the same DOX concentration had stronger red fluorescence, and the green fluorescence intensity was far less than that of the DOX group, indicating that the mitochondrial membrane potential after PDA(DOX) treatment was consistent with that of normal cells.

**Fig. 7:**
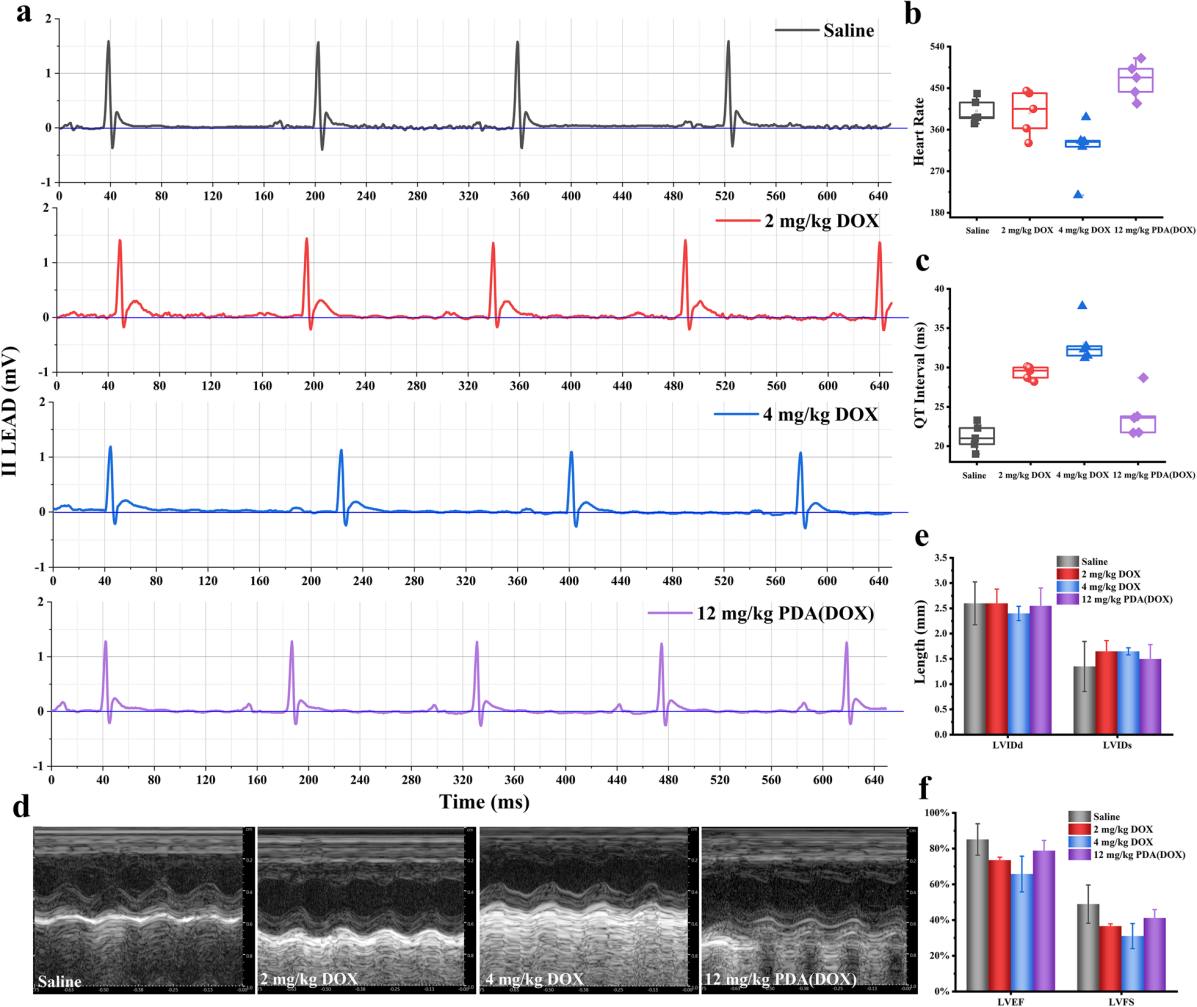
In vivo mitigation of DIC effectiveness evaluation with electrocardiogram and echocardiography. **a** The electrocardiogram of mice in different chemotherapy groups. **b**, **c** The heart rate and QT interval of mice in different groups by electrocardiogram inspections. **d** The echocardiography of mice in different chemotherapy groups. **e**, **f** The left ventricular inner diameter diastole (LVIDd), left ventricular inner diameter systole (LVIDs), left ventricular ejection fraction (LVEF), and LV fractional shortening (LVFS) of mice in different groups by echocardiography inspections

During chemotherapy, the 4 mg/kg DOX group mice exhibited symptoms such as rough fur, delayed response, reduced food intake, and delayed movement. Furthermore, before the end of chemotherapy, an electrocardiogram and echocardiography were performed on each group of mice to evaluate the effect of PDA on attenuating DIC. As shown in Fig. 7a–c, the heart rate and QT interval of mice were calculated using electrocardiogram data from different groups of mice. The results showed that the heart rate of mice in the 4 mg/kg DOX group was significantly reduced. And the QT interval of mice treated with two different DOX concentrations chemotherapy groups was particularly prolonged. On the contrary, the heart rate and QT interval of the 12 mg/kg PDA(DOX) group were similar to those of the control group. Meanwhile, echocardiographic indicators also showed a decrease in LVIDd in the 4 mg/kg DOX group, consistent with the 2 mg/kg DOX group, with a significant increase in LVIDs (Fig. 7d, e). As a result, there was a significant decrease in LVEF and LVFS in both DOX groups (Fig. 7f). However, the results of four echocardiographic indicators in the 12 mg/kg PDA (DOX) group were consistent with those in the control group, indicating that PDA nanoenzyme drug carriers can effectively alleviate DIC.

**Fig. 8:**
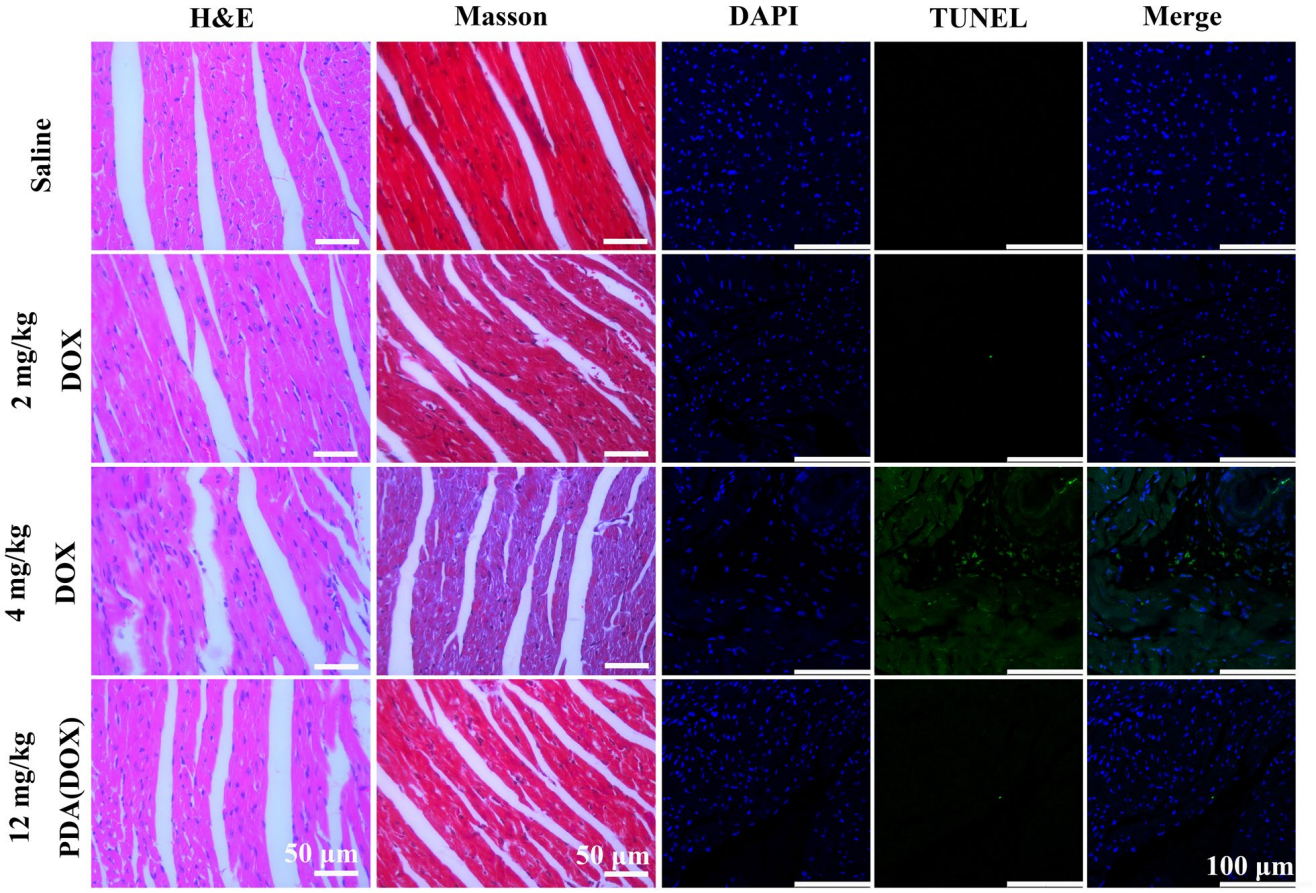
H&E, Masson’s trichrome, DAPI, and TUNEL staining images of mice hearts after different chemotherapy treatments for 14 days

After eight intravenous injections over 14 days, the mice were sacrificed, and hearts in different groups were collected for further pathological analysis (Fig. 8). The H&E staining slices of mice hearts showed that the 2 and 4 mg/kg DOX groups exhibited myocardial cell hypertrophy, cytoplasmic vacuolization, and irregular muscle fiber texture orientation. In addition, myocardial fiber breakage was observed in the 4 mg/kg DOX group. The hypertrophy of myocardial cells in the PDA(DOX) group was significantly alleviated compared to the two different dosages of DOX groups. The morphology of myocardial cells was normal, the cytoplasm staining was uniform, and the texture of muscle fibers was regular and without abnormalities. Moreover, Masson’s trichrome staining was performed to estimate cardiac fibrosis. Figure 8 and Additional file 1: Figure S15 showed that collagen fibers stained blue were observed after injection of 2 mg/kg and 4 mg/kg DOX compared with the saline group. Heart sections treated with PDA(DOX) observed fewer collagen fibers conversely. To be noticed, TUNEL staining takes on the more obvious comparison. The green fluorescence signal in the PDA(DOX) group was significantly lower than that of the 4 mg/kg DOX group. And when treated with PDA(DOX), H&E staining showed a smaller lesion area.

In addition, as shown in Fig. 5f, among the four indicators of the myocardial enzyme spectrum of biochemical blood detection, the AST, CK, and CKMB indexes of the 4 mg/kg DOX group were higher than those in other groups. In contrast, the LDH index was lower of the two different concentrations of DOX groups, indicating that the myocardial damage caused by DOX occurred during chemotherapy. However, compared with the control group, there were no significant differences in all the myocardial enzyme profiles in the PDA(DOX) group.

In summary, PDA(DOX) can effectively alleviate DOX-induced myocardial injury both in vitro and in vivo. Therefore, this technology will further expand the application scope of DOX in tumor chemotherapy, providing the scientific basis for achieving safe and efficient use of DOX.

### *Acute toxic profiles of PDA (DOX) *via* intravenous administration*

**Fig. 9:**
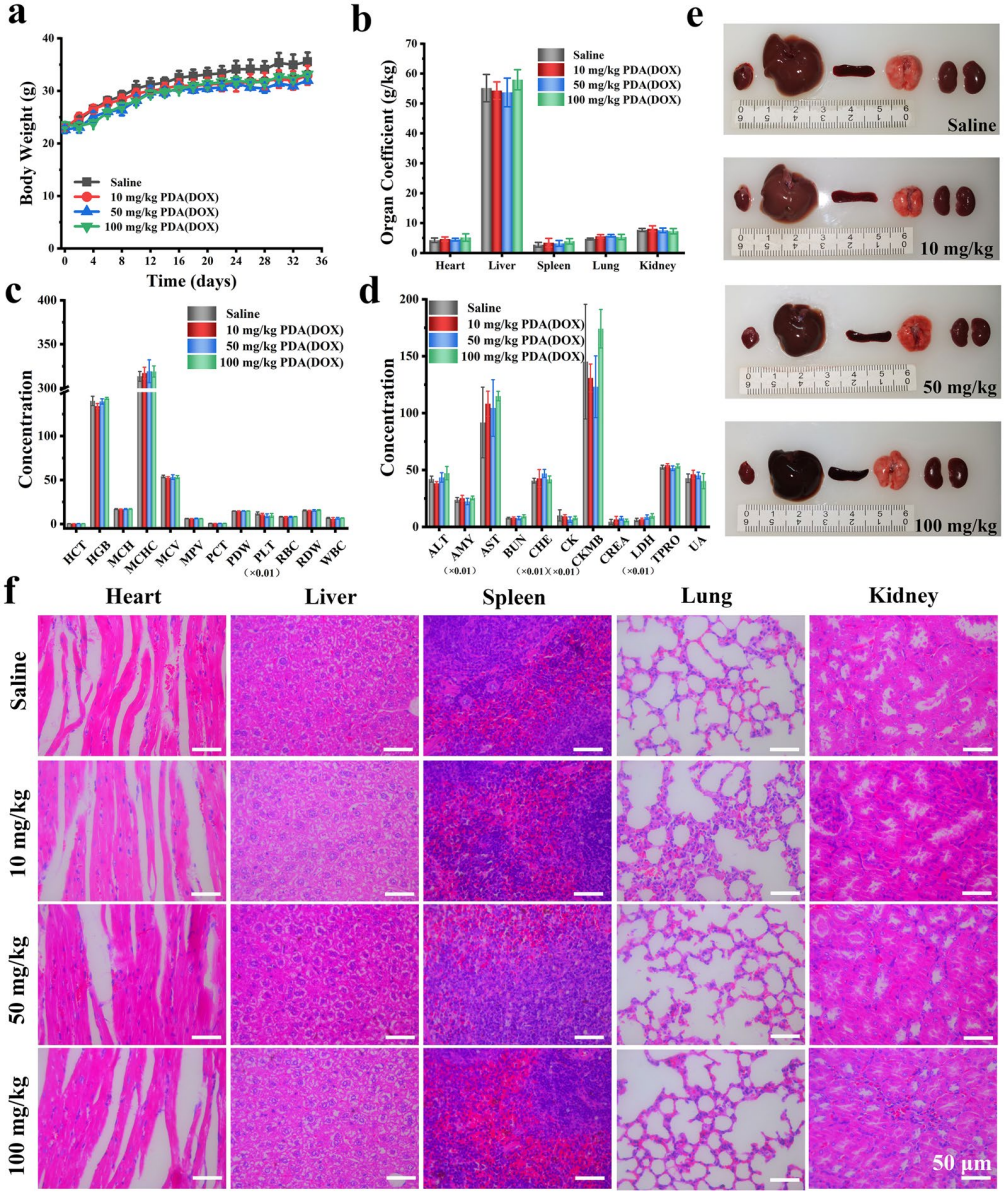
Acute toxic profiles of PDA(DOX) nanoparticles via intravenous administration. **a** Body weight of mice treated with PDA(DOX) within 35 days; **b** Organ coefficient of mice treated with PDA(DOX) after 35 days; **c** Blood routine test of mice after sacrifice; **d** Serum biochemical indices mice after sacrifice; **e** Major organs photographs of mice treated with PDA(DOX); **f** H&E staining images of mice major organs

To confirm the safety of PDA (DOX), healthy ICR male mice were randomly divided into four groups, intravenously injected with PDA(DOX) at different concentrations of 0, 10, 50, and 100 mg/kg. Over the next 35 days, mice in each PDA(DOX) dose group behaved normally, and no deaths occurred compared to the control group. Figure 9a shows that during the toxicity test, the weight of mice in each group increased steadily without significant difference. On the 35th day, all of the mice were sacrificed, followed by the collection of blood samples and primary organs (heart, liver, spleen, lung, and kidneys). We found that the liver and spleen had different degrees of color changes in the medium and high concentration groups because PDA(DOX) accumulated in the two organs through blood circulation (Fig. 9e). In addition, the results of organ coefficient (Fig. 9b) and H&E staining (Fig. 9f) showed no noticeable damage in kidney tissue from the PDA(DOX) groups, indicating renal impairment was almost nonexistent.

Furthermore, H&E staining of other primary organs revealed no evident damage (Fig. 9f). and the routine blood tests and serum biochemical indices in the PDA (DOX)-treated groups were within normal ranges (Fig. 9c, d). Above all, PDA (DOX) has been proven to have excellent biocompatibility and low toxicity, so that can be used safely below 100 mg/kg.

## Conclusion

In this study, we reported a nanomedicine PDA(DOX) via the one-pot method under mild conditions based on PDA nanoparticles having plentiful aromatic rings on their surface. PDA(DOX) is an outstanding multifunctional drug in breast cancer treatment. PDA(DOX) could play a therapeutic role because of its good photothermal properties and chemotherapy effect. Upon laser irradiation, the excellent photothermal conversion efficiency of PDA(DOX) can quickly convert light energy into thermal energy, increase the temperature of the tumor site, and achieve thermal ablation. Moreover, under the acidic microenvironment of the tumor, PDA(DOX) can effectively release DOX to achieve tumor chemotherapy. PDA nanozyme further solved the problem of DIC. In vitro and in vivo experiments revealed that PDA(DOX) can alleviate the damage of DOX to myocardial cells by scavenging ROS and reducing oxidative stress levels. Consequently, with the antioxidation property and chemo-photothermal capability, PDA(DOX) nanoplatform will be proposed as a prominent candidate drug for cancer treatment.

### Supplementary Information

**Additional file 1: Figure S1.** The TEM images and size distributions of PDA nanoparticles. **Figure S2.** The visible-NIR absorbance spectra of PDA, DOX, and PDA(DOX). **Figure S3.** The standard curves between the mass concentration of PDA-i, PDA(DOX), and DOX. **Figure S4.** The temperature rise curves of PDA-*i* and PDA(DOX) dispersions with different PDA concentrations. **Table S1**. The Δ*T*, I, Q_dis_, τ_s_, *h*s, and A_808 nm_ of PDA and PDA(DOX) nanoparticles for calculating photothermal conversion efficiency η. **Figure S5.** Temperature rise and fall curves of PDA-*i* and PDA(DOX) and liner time data versus—lnθ from the cooling of PDA-*i* or PDA(DOX) for obtaining the τ_s_. **Table S2.** The D, *A*_808 nm_, *V*_NC_, and C_wt_ of PDA and PDA(DOX) nanoparticles for calculating ε_808 nm_, σ_808 nm_, and Q_808 nm_. **Figure S6.** The cytotoxicity of various concentrations of DOX on H9C2 cells. **Figure S7.** Confocal microscope images of H9C2 and 4T1 cells uptake DOX and PDA(DOX) for 2 h with different concentrations. **Figure S8.** Confocal microscope images of H9C2 and 4T1 cells uptake DOX and PDA(DOX) for 4 h with different concentrations. **Figure S9.** Tumor weight of the excised tumors of mice after sacrifice and body weights of 4T1 tumor-bearing mice within 21 days after synergetic chemo-photothermal therapy. **Figure S10.** Micrographs of H&E-stained major organ slices from mice with synergetic chemo-photothermal therapy were collected after 21 days. **Figure S11.** 4T1 tumor-bearing mice images on the 0, 7, and 14 days after different treatments and photographs of the major organs of mice after sacrifice. **Figure S12.** Organ Coefficient of chemotherapy mice after sacrifice within 14 days after different treatments. **Figure S13.** In vivo synergetic chemo-photothermal therapy of 4T1 tumor-bearing mice irradiated by 808 nm laser at a laser power of 0.8 W/cm^2^ for 5 min, and then implement chemotherapy. **Figure S14.** 4T1 tumor-bearing mice images after different treatments, and the tumor growth curves from individual 4T1 tumor-bearing Balb/c mice in different treatment groups (NIR: 0.8 W/cm^2^ for 5 min). **Figure S15.** H&E and Masson-staining micrographs of major organ slices from different chemotherapy groups after sacrifice within 14 days.

## Data Availability

All data are available in the main text or the supplementary materials and are available from the corresponding authors upon reasonable request.
