# Supplementary material for: A Versatile PDA(DOX) Nanoplatform for Chemo-Photothermal Synergistic Therapy against Breast Cancer and Attenuated Doxorubicin-Induced Cardiotoxicity
Source: J Nanobiotechnology. 2023 Sep 21;21:338. doi: 10.1186/s12951-023-02072-1 (PMC10512561; doi:10.1186/s12951-023-02072-1)
Supplement: Supplementary file 1 — Additional file 1: Figure S1. The TEM images and size distributions of PDA nanoparticles. Figure S2. The visible-NIR absorbance spectra of PDA, DOX, and PDA(DOX). Figure S3. The standard curves between the mass concentration of PDA-i, PDA(DOX), and DOX. Figure S4. The temperature rise curves of PDA-i and PDA(DOX) dispersions with different PDA concentrations. Table S1. The ΔT, I, Qdis, τs, hs, and A808 nm of PDA and PDA(DOX) nanoparticles for calculating photothermal conversion efficiency η. Figure S5. Temperature rise and fall curves of PDA-i and PDA(DOX) and liner time data versus—lnθ from the cooling of PDA-i or PDA(DOX) for obtaining the τs. Table S2. The D, A808 nm, VNC, and Cwt of PDA and PDA(DOX) nanoparticles for calculating ε808 nm, σ808 nm, and Q808 nm. Figure S6. The cytotoxicity of various concentrations of DOX on H9C2 cells. Figure S7. Confocal microscope images of H9C2 and 4T1 cells uptake DOX and PDA(DOX) for 2 h with different concentrations. Figure S8. Confocal microscope images of H9C2 and 4T1 cells uptake DOX and PDA(DOX) for 4 h with different concentrations. Figure S9. Tumor weight of the excised tumors of mice after sacrifice and body weights of 4T1 tumor-bearing mice within 21 days after synergetic chemo-photothermal therapy. Figure S10. Micrographs of H&E-stained major organ slices from mice with synergetic chemo-photothermal therapy were collected after 21 days. Figure S11. 4T1 tumor-bearing mice images on the 0, 7, and 14 days after different treatments and photographs of the major organs of mice after sacrifice. Figure S12. Organ Coefficient of chemotherapy mice after sacrifice within 14 days after different treatments. Figure S13. In vivo synergetic chemo-photothermal therapy of 4T1 tumor-bearing mice irradiated by 808 nm laser at a laser power of 0.8 W/cm2 for 5 min, and then implement chemotherapy. Figure S14. 4T1 tumor-bearing mice images after different treatments, and the tumor growth curves from individual 4T1 tumor-bearing Balb/c m [file 12951_2023_2072_MOESM1_ESM.docx]

**Additional Information**

**A Versatile PDA(DOX) Nanoplatform for Chemo-Photothermal Synergistic Therapy against Breast Cancer and Attenuated Doxorubicin-Induced Cardiotoxicity**

Siqi Geng ^1, 2^, Qiang Feng ^2^, Chujie Wang ^2^, Ying Li ^2^, Jiaying Qin ^2^, Mingsheng Hou ^3^, Jiedong Zhou ^2^, Xiaoyu Pan ^2^,

Fei Xu ^4^, Baoru Fang ^1, 2^, Ke Wang ^1, 2^, Zhangsen Yu ^2^, ^1*^

*^1^ School of Life and Environmental Sciences, Shaoxing University, Shaoxing City, Zhejiang Province, 312000, P. R. China*

*^2^ Laboratory of Nanomedicine, Medical Science Research Center, School of Medicine, Shaoxing University, Shaoxing City, Zhejiang Province, 312000, P. R. China*

*^3^ Department of Pathology, Shaoxing Hospital of Traditional Chinese Medicine, Shaoxing City, Zhejiang Province, 312000, P. R. China*

*^4^ Department of Ultrasound, Affiliated Hospital of Shaoxing University, Shaoxing City, Zhejiang Province, 312000, P. R. China*

^*^ *Corresponding author:* [yzs@usx.edu.cn](mailto:yzs@usx.edu.cn)

**S1. Experimental Section**

**S1.1. Materials, cell lines, and animal models**

Dopamine hydrochloride (98%), Doxorubicin hydrochloride (98%), Ammonium hydroxide solution (28 wt %), and Thiazoly blue tetrazolium bromide (98%) were purchased from Aladdin Ltd. (Shanghai, China). The total superoxide dismutase assay kit with WST-8, total antioxidant capacity assay kit with a rapid ABTS method, enhanced cell mitochondrial membrane potential assay kit with JC-1, one-step TUNEL apoptosis assay kit, DAPI staining solution, cell plasma membrane staining kit with DiO, 4% Paraformaldehyde Fix Solution, and regenerated cellulose dialysis membranes (MWCO = 3.5 kDa) were obtained from Beyotime Biotechnology (Jiangsu, China). The reactive oxygen species assay kit, Masson’s trichrome stain kit, and Annexin V-FITC/PI Apoptosis Detection Kit were the products of Solarbio Science & Technology Co. Ltd. (Beijing, China). RPMI 1640 medium, DMEM medium (high glucose), fetal bovine serum (FBS), penicillin-streptomycin-L-glutamine, and trypsin-EDTA (0.25%) were bought from Gibco Laboratories (Grand Island, NY, USA). The MCF-10A-specific medium was obtained from Procell (Wuhan, China). 4T1 murine breast cancer, H9C2 rat cardiomyocyte, and MCF-10A human mammary epithelial cell lines were purchased from the Shanghai Cell Bank of the Chinese Academy of Sciences. Balb/c mice (permission number: 20220711ABzz0100999763) and ICR mice (permission number: 20220624Abzz01009994376) were bought from Hangzhou Qizhen Experimental Animal Technology Co., Ltd. (Hangzhou, China; production license number: SCXK (Zhe) 2022-0005). All animal procedures described below were performed under the protocol approved by the Animal Care Research Committee of Shaoxing University. Animal experiments were conducted under pathogen-free conditions in the First Affiliated Hospital of Shaoxing University (Permit No. SYXK (Zhe) 2017-0007). All the materials mentioned were analytical grade and used directly without further purification.

**S1.2. Synthesis and characterization of PDA(DOX) nanoplatform**

PDA nanoparticles were self-assembled by oxypolymerization[1], which were synthesized by oxidation and dopamine self-polymerization in the mixed solution, including ammonia, ethanol, and deionized water. Briefly, PDA was synthesized according to the previously reported hydrothermal protocol, whereas the amount of ammonia added to the reaction system was adjusted[2]. Different contents of aqueous ammonia solution (0.6, 0.9, 1.2, 1.5, 1.8, 2.1, and 2.4 mL) were added into a mixture of 24 mL ethanol and 54 mL deionized water maintained at 30°C, respectively. After 30 min stirring, 300 mg of dopamine was dissolved in 6 mL deionized water, and then continue the oxidative polymerization reaction with mildly magnetic stirring for 24 h under 30°C. As a result, the color of the solution turns from pale brown to dark brown gradually. PDA was obtained by centrifugation, washed with deionized water several times, and dispersed in deionized water.

PDA(DOX) was prepared by dissolving PDA and DOX in 2 mL PBS at different mass ratios (m_PDA_ : m_DOX_ = 1:0.25, 1:0.5, 1:0.75, 1:1, and 1:2) under magnetic stirring at room temperature for 24 h. After that, PDA(DOX) was collected by centrifugation at 12500 rpm for 10 min and washed with deionized water several times until the supernatant was completely colorless. Then, PDA(DOX) was dispersed in PBS for further investigation. The supernatant was collected to detect absorbance at 488 nm by the microplate reader (SpectraMax i3, Molecular Devices, USA), which was used to calculate the DOX loading efficiency and capacity. Moreover, PDA and different PDA(DOX) mass ratios were diluted to 50 μg/mL, an excitation wavelength of 488 nm, to detect the fluorescence intensity between 520 to 700 nm using a fluorescence spectrophotometer. The calculation formulas of the DOX loading efficiency and capacity are as follows:

$$Loading\text{ }efficiency\text{ }=\text{ }\frac{M_{DOX}\text{ }-\text{ }m_{DOX}}{M_{DOX}}\text{ }\times\text{ }100\%$$

$$Loading\text{ }capacity\text{ }=\text{ }\frac{M_{DOX}\text{ }-\text{ }m_{DOX}}{M_{PDA}\text{ }+\text{ }(M_{DOX}\text{ }-\text{ }m_{DOX})}\text{ }\times\text{ }100\%$$

Where *M_DOX_* and *M_PDA_* are the total DOX and PDA mass in the reaction solution, respectively, *m_DOX_* is the mass of DOX in the supernatant collected by centrifugation. After comparison and analysis, the mass ratio of 1:0.75 was chosen to synthesize PDA(DOX) for future use. Finally, we have obtained sufficient PDA(DOX) with a loading capacity of 33.6% and a loading efficiency of 67.5% for cell and animal experiments.

The PDA morphology was characterized with Transmission electron microscopy (JEM-2100F, Japan). And the average and standard deviation of particle size with 100 PDA nanoparticles were calculated by Nano Measurer 1.2 software. A laser particle sizer (Nano ZS, Malvern Instruments, UK) determined the dynamic light scattering and Zeta potential of PDA and PDA(DOX). Fluorescence spectra were obtained by Fluorescence spectrometer (RF-6000, SHIMADZU). A UV-Vis spectrometer (UV-1900, Shimadzu, Japan) recorded the UV-Vis-NIR absorbance spectra.

**S1.3. Photothermal performance evaluation**

PDA and PDA(DOX) were diluted to 50 μg/mL (the concentration of PDA) and conducted with a UV-1900 spectrometer to determine the absorption spectra. The photothermal performances of PDA and PDA(DOX) nanoparticles were evaluated *via* the temperature rise curve, photothermal conversion efficiency, and photothermal stability. First, PDA and PDA(DOX) were aqueous suspensions diluted at different concentrations (PDA concentrations were 50, 100, 200, 300, and 400 μg/mL). Then, 1 mL PDA or PDA(DOX) was held in a quartz cuvette irradiated with an 808 nm NIR laser at a laser power of 1.5 W for 300 s. The temperature was measured throughout the process, and thermal images were recorded by the thermal imaging system (PI400, Optris, Germany). Furthermore, under the same condition, 50 μg/mL PDA and PDA(DOX) samples were heated to thermal equilibrium and turned off the laser. Then the suspensions were naturally cooled to room temperature. The temperature variation in the cooling process was recorded to calculate photothermal conversion efficiency (η). Finally, photothermal stability was evaluated through five laser cycles on or off. That is, the 1 mL sample was irradiated under 808 nm laser at 1.5 W for 5 min, followed by natural cooling for 5 min.

**S1.4. Multienzyme-like activity of PDA(DOX) nanoplatform**

**Superoxide dismutase-like activity.** The superoxide dismutase activity of the PDA nanozyme was analyzed by the SOD assay kit with WST-8. 20 μL PDA or PDA(DOX) dispersions were added to 160 μL WST-8/enzyme working solution (the PDA final concentrations were 0.025, 0.05, 0.1, and 0.2 mg mL^-1^). After adding 20 μL reaction starting solution and incubating at 37°C for 30 min, the absorbance at 450 nm and 650 nm were obtained through the microplate reader (SpectraMax i3, Molecular Devices, USA), which is used to calculate the inhibition rate of SOD.

**Total antioxidant capacity.** A total antioxidant capacity assay kit with a rapid ABTS method was used to assess PDA and PDA(DOX). The PDA concentrations of different nanoparticles were 3.125, 6.25, 12.5, 18.75, and 25 μg/mL. First, a 10 μL sample was added to a 20 μL peroxidase working solution. After that, each mixed solution was incubated with 170 μL ABTS working solution at room temperature for 6 min. The microplate reader determined the absorbance at 414 nm of the solution. And the ABTS radical (ABTS•^+^) scavenging abilities were calculated as follows: ABTS•^+^ scavenging ratio (%) = (1 - A_sample_ / A_control_) × 100. A_control_ is the absorbance value of a standard solution without any radical scavengers, and A_sample_ is the absorbance value after the reaction with the radical scavengers, respectively.

**S1.5. *In vitro* DOX release experiments**

The dialysis method was used to assess the DOX release kinetics with the stimulation of pH and temperature, set four groups as follows: (1) pH = 7.4, 25°C (2) pH = 7.4, 37°C (3) pH = 5.7, 25°C (4) pH = 5.7, 37°C. 2 mL of PDA(DOX) at a concentration of 5 mg mL^-1^ were packaged in dialysis bags (MWCO = 3.5 kDa), immersed within 250 mL PBS under magnetic stirring at the corresponding temperature. In addition, 300 μL of supernatant was taken out at different time points, and the same amount of fresh PBS was added. The DOX concentration in the supernatant was measured by fluorescence intensity at 588 nm. Finally, the cumulative release percentages of DOX were calculated to evaluate the release kinetics.

**S1.6. Cell experiments**

**Cell culture.** There were three different types of cell lines, Murine breast cancer 4T1 cell line (4T1), rat cardiomyocyte cell line (H9C2), and human mammary epithelial cell line (MCF 10A). 4T1 and H9C2 cells were cultivated in the RPMI-1640 medium and high glucose DMEM medium, respectively, containing 10% fetal bovine serum and 1% penicillin/streptomycin at 37°C in 5% CO_2_. MCF 10A cells were cultured in the MCF 10A-specific medium.

**Cytotoxicity assays.** Cytotoxicity assays of PDA(DOX) were analyzed on 4T1, H9C2, and MCF 10A. First, the relative cell activities were determined through the standard MTT assays. Then, cells were incubated in 96-well plates at 10^4^ cells per well and incubated overnight at 37°C under a humidified 5% CO_2_. Next, PDA(DOX) and DOX with different concentrations (the final measured DOX concentrations were 6.3, 12.6, 25.2, and 50.4 μg/mL) were added into wells for 24 h. After that, the cells were washed with PBS twice and treated with MTT to allow formazan dye formation. Then, 4 h later, the purple formazan in each well was dissolved with DMSO for 10 min. Finally, the microplate reader (SpectraMax i3, Molecular Devices, USA) measured the absorption of formazan at 490 nm.

**Cellular uptake observation.** Cellular uptake of DOX and PDA(DOX) was detected on 4T1 and H9C2 cells. Cells were seeded into a four-well chambered cover glass at a density of 6 × 10^4^ per well. DOX or PDA(DOX) was added to wells to make the final concentrations 0.025, 0.05, 0.1, and 0.2 μg/mL (in terms of DOX) after ensuring that the cells were adherent. Then, 2 h or 4 h later, PBS was washed three times, and 4% paraformaldehyde fix solution was added into each well for 30 min. After PBS washing, DiO staining solution was added for incubating at 37°C protected from light for 10 min. Then, DAPI staining solution was added for dye at room temperature for 4 min. Finally, PBS was washed thrice for 4 minutes each, and fluorescence images were taken through a laser scanning confocal microscope (Zeiss, LSM 900).

**Apoptosis assay.** Annexin V-FITC/PI apoptosis detection kit was used to evaluate 4T1. First, cells were seeded into a 6-well plate at a density of 4 × 10^5^ per well. After the cell adhesion, DOX (0.05, 0.10 μg/mL) or PDA(DOX) (0.15, 0.30 μg/mL) was added into the wells. Then, 24 h later, cells were collected, washed with pre-chilled PBS, and dispersed into the 1× binding buffer, respectively. Finally, 200 μL cells were stained with Annexin V-FITC (10 μL) and PI (5 μL) for flow cytometry (Beckman Coulter, Cytoflex, USA) analysis.

**S1.7. *In vitro* synergistic chemo-photothermal therapy effect**

The synergistic chemo-photothermal therapy effect of PDA(DOX) *in vitro* was evaluated on 4T1 cells. First, the 4T1 cells were cultured in 96-well plates at 10^4^ cells per well overnight at 37 °C. The cells were divided into five groups: control, only laser, PDA + laser, PDA(DOX) + laser, and PDA(DOX). Then, cells were incubated with corresponding drugs for 4 h. The drugs had the same PDA concentrations, 25, 50, and 100 μg/mL, respectively. Therefore, the PDA(DOX) concentrations were 37.5, 75, and 150 μg/mL. After that, cells in laser groups were irradiated with an 808 nm laser at a laser power of 1.5 W for 5 min. Then, cells continued to incubate for 20 h and were washed with PBS twice. Finally, the standard MTT assay was carried out to determine the relative cell viability after synergistic chemo-photothermal treatment.

**S1.8. Oxidative stress of H9C2 cell evaluation**

DCFH-DA staining was used to detect intracellular ROS levels. First, H9C2 cells were seeded into a four-well chambered cover glass at a density of 6 × 10^4^ per well, cultivating overnight until adhesion. Then, DOX or PDA(DOX) was added into wells to make the final concentrations of 0.025, 0.05, and 0.1 μg/mL (in terms of DOX). The treated cells were cultured for 24 h, and then the reactive oxygen species assay kit was adopted to evaluate the level of ROS in cells by fluorescence of DCF. After DCFH incubation for 20 minutes, cells were washed three times by DMEM. Finally, fluorescence images were taken through the laser-scanning confocal microscope.

An enhanced mitochondrial membrane potential assay kit with JC-1 was carried out to test mitochondrial membrane potentiality. First, H9C2 cells (6 × 10^4^ per well) were seeded overnight in a four-well chambered cover glass until adhesion[3]. Then, DOX or PDA(DOX) was added into wells with different DOX concentrations of 0.025, 0.05, and 0.1 μg/mL. After 24 h incubation, the cell medium was absorbed, and cells were incubated with JC-1 staining solution for 20 min at 37 °C. After that, washed twice with JC-1 dye buffer and observed under a laser scanning confocal microscope.

**S1.9. Tumor model**

The murine breast cancer cell line 4T1 was injected into female Balb/c mice (4-6 weeks old) to construct a subcutaneous breast cancer model. 10^6^ 4T1 cells were suspended in 50 μL of serum-free cell medium and subcutaneously injected in female Balb/c mice, respectively. The mice were used for further experiments when the tumor volume had grown to 50 - 60 mm^3^. The tumor sizes were measured by a digital caliper and calculated as the volume. Tumor volume was calculated as V = W^2^ × L/2, where W and L are the tumor width and length, respectively. The tumor growth inhibition index (TGI) was calculated as follows: $TGI\text{ }=\text{ }1\text{ }-\text{ }V_{T}/V_{C}$. *V_T_* and *V_C_* are the mean volumes of treated and controlled tumors.

**S1.10. *In vivo* chemo-photothermal synergistic therapy studies**

The tumor-bearing mice were randomly divided into six groups (n = 6 for each group): saline (I), saline + laser (II), PDA + laser (III), DOX (IV), PDA(DOX) (V), and PDA(DOX) + laser (VI), intratumorally injected with 50 μL drug (PDA 2 mg mL^-1^, PDA(DOX) 3 mg mL^-1^, and DOX 1 mg mL^-1^) per mouse. Then, 4 h of drug diffusion later, the tumor of groups II, III, and VI received 808 nm laser radiation at a power of 2.0 W cm^-2^ for 5 min. The thermal images visually recorded the temperature change. The tumor sizes and body weight were measured every other day after treatment. On the 21^st^ day of the synergistic therapy, all the mice were sacrificed, and the blood was used to detect routine blood tests and biochemical studies. And the main organs (heart, liver, spleen, lung, and kidney) and tumors were collected for organ coefficient and histology analysis.

**S1.11. Electrocardiogram and echocardiography for cardiac functions evaluation**

For studying the efficacy of PDA(DOX) on attenuating DIC, the tumor-bearing mice were randomly divided into five groups (n = 8 for each group): saline, 2 mg/kg DOX, 4 mg/kg DOX, and 12 mg/kg PDA(DOX). When the tumor volume reached 50 mm^3^, it injected corresponding drugs intravenously. During 21 days, chemotherapy drugs were injected intravenously eight times.

Cardiac functions were evaluated by electrocardiogram (EGC) and echocardiography. The tumor-bearing mice were anesthetized with isoflurane using a small animal anesthesia device (R500IE, RWD, Shenzhen, China). Two acupuncture needle electrodes were inserted under the skin of the right front paw (negative electrode) and left hind leg paws (positive electrode), respectively. A biological signal analytical system (BL-420N, Taimeng software, Chengdu, China) recorded standard lead II electrocardiogram signals. In addition, the ECG pattern changes of the QT interval were determined. And the heart rate was calculated by spectral analysis of the R-R interval from the ECG signal. Cardiac echocardiography was assessed using the GE LOGIQ E10 system equipped with an 18 MHz linear ultrasonic transducer (GE L8-18i linear transducer). The morpho-functional parameters for cardiac function evaluation include left ventricular inner diameter diastole (LVIDd), left ventricular inner diameter systole (LVIDs), left ventricular ejection fraction (LVEF), and LV fractional shortening (LVFS).

**S1.12. *In vivo* acute toxicity assessment**

Male ICR mice (4 - 6 weeks) were prepared to evaluate the acute toxicity of PDA(DOX). The mice were randomly divided into four groups (n = 6 for each group) as follows, saline, 10 mg/kg PDA(DOX), 50 mg/kg PDA(DOX), and 100 mg/kg PDA(DOX). Different PDA(DOX) doses were injected intravenously on the first day. In 35 days, the mice's body weight and physical and mental health conditions were assessed every other day. On the last day, blood was collected from mice's eyeballs for routine blood tests and biochemical studies. In addition, the main organs, including the heart, liver, spleen, lung, and kidney, were taken to calculate organ coefficient and hematoxylin and eosin (H&E) staining.

**S1.13. Histology analysis (H&E, Masson, and TUNEL stain)**

Histological changes in main organs tissue were examined by H&E staining. And the cardiac tissues of the mice in the chemotherapy groups were further analyzed by Masson staining and TUNEL staining. Paraffin-embedded organ tissues were cut into 4 μm thick sections. For H&E staining, the tissue sections were deparaffinized by xylene and then stained with hematoxylin and eosin at room temperature. For Masson staining, heart histological sections were dewaxed and stained with Weigert's iron hematoxylin solution, ponceau-acid fuchsin solution, and blue aniline solution turn. Finally, the slices were dehydrated and sealed. For the TUNEL staining, heart tissue sections were incubated with proteinase K (free DNase) for 20 min after being dewaxed to distilled water. After washing with PBS, incubated with TUNEL detection solution at 37°C in the dark for 1 h, stained with DAPI for 5 min. Finally, the antifade mounting medium was dropped into heart tissue sections for further viewing.

**S1.14. Statistical analysis**

All experimental data were expressed as the mean ± standard deviation and analyzed by student t-test. One-way ANOVA followed by Dunnett’s multiple comparison tests was used to compare the groups. Differences were considered statistically significant at **p* < 0.05, ***p* < 0.01, and ****p* < 0.001.

**S2. Theoretical Calculation and Analysis of photothermal performance results**

To elucidate the effect of PDA sizes on their photothermal properties, we further calculated the photothermal conversion efficiency, absorption cross-section, absorption efficiency factor, and molar absorption coefficient of PDA nanoparticles with different particle sizes.

**S2.1. Photothermal conversion efficiency**

Following Roper’s report and Lambert-Beer law, the 808 nm photothermal conversion efficiency (η) of PDA nanoparticles can be determined by Equation S1:

$\eta=\frac{hS(T_{\max}-T_{surr})-Q_{dis}}{I(1-10^{-A_{808\text{ }nm}})}$ (S1)

Where η is the photothermal conversion efficiency from incident 808 nm laser energy to thermal energy, *h* is the heat transfer coefficient, *S* is the surface area of the container, *T_max_* is the equilibrium temperature, *T_surr_* is the ambient temperature of the surroundings, *Q_dis_* is the baseline energy inputted by the sample cell, *I* is the incident 808 nm laser power, and *A_808 nm_* is the absorbance of the PDA nanoparticles in 808 nm. Among them, the data of *S*, *T_max_*, *T_surr_*, *I,* and *A_808 nm_* can be obtained by direct measurement. However, the data of *hS* and *Q_dis_* were calculated according to the specific implementation of the photothermal performance test experiment. Based on the experimental data obtained from the thermal equilibrium conditions, the value of *h*S can be calculated. In addition, the *Q_dis_* expresses heat dissipated from the 808 nm light absorbed by the quartz sample cell. It was measured by a quartz cuvette cell containing pure water without the PDA nanoparticles. The specific heat capacity of the water and quartz curette were 4.2 and 0.892 J g^-1^ K^-1^), respectively.

**S2.2. Absorption cross-section**

For the dilute dispersion of small nanoparticles, the extinction cross-section is determined by absorption. The absorption cross-section of the PDA nanoparticles can be calculated by the Beer-Lambert law as Equation S2.

$\sigma_{808\text{ nm}}\text{ = }\frac{3.071\pi\rho r^{3}\text{A}_{\text{808 nm}}}{LC_{wt}}$ (S2)

Where ρ is the mass density of PDA (1.247 g mL^-1^), *r* is the radius of PDA nanoparticles, *A_808 nm_* is the absorbance of the PDA nanoparticles in 808 nm, *L* is the light path length (1 cm), and *C_wt_* is the mass concentration of PDA (50 μg/mL).

**S2.3. Absorption efficiency factor**

The absorption efficiency factor (Q_808 nm_) was calculated by normalizing the optical absorption cross-sections $\sigma_{808\text{ nm}}$ against the physical cross-section of the nanoparticle using Equation S3.

$Q_{808\text{ nm}}\text{ = }\frac{\sigma_{808\text{ }nm}}{\pi r^{2}}\text{ = }\frac{3.071\rho r\text{A}_{\text{808 nm}}}{LC_{wt}}$ (S3)

**S2.4. Molar absorption coefficient**

According to the Lambert-Beer law, the formula for the molar absorption coefficient in the 808 nm (*ε_808 nm_*) of the PDA nanoparticles is shown in Equation S4.

$\varepsilon_{808\text{ nm}}\text{ = }\frac{4\pi A_{\text{808 nm}}\rho N_{A}r^{3}}{3LC_{wt}}$ (S4)

Where *A_808 nm_* is the absorbance at the wavelength of 808 nm, ρ is the mass density of PDA (1.247 g mL^-1^), *N_A_* is the Avogadro’s constant (6.02×10^23^ mol^-1^), *r* is the radius of PDA nanoparticles, *L* is the light path length (1 cm), and *C_wt_* is the mass concentration of PDA (50 μg/mL).

**References**

1. Hong S, NaYS, Choi S, Song IT, Kim WY, Lee H. Non-covalent self-assembly and covalent polymerization co-contribute to polydopamine formation. *Adv Funct Mater.* **2012**;22(22):4711-4717.

2. Liu Y, Ai K, Liu J, Deng M, He Y, Lu L. Dopamine-melanin colloidal nanospheres: an efficient near-infrared photothermal therapeutic agent for in vivo cancer therapy. *Adv Mater*. **2013**;25(9):1353-9.

3 Chen X, Tai L, Gao J, Qian J, Zhang M, Li B, Xie C, Lu L, Lu W, Lu W. A stapled peptide antagonist of MDM2 carried by polymeric micelles sensitizes glioblastoma to temozolomide treatment through p53 activation. *J Control Release*. **2015**;218:29-35.

**S3. Additional Figures and Calculation Results**


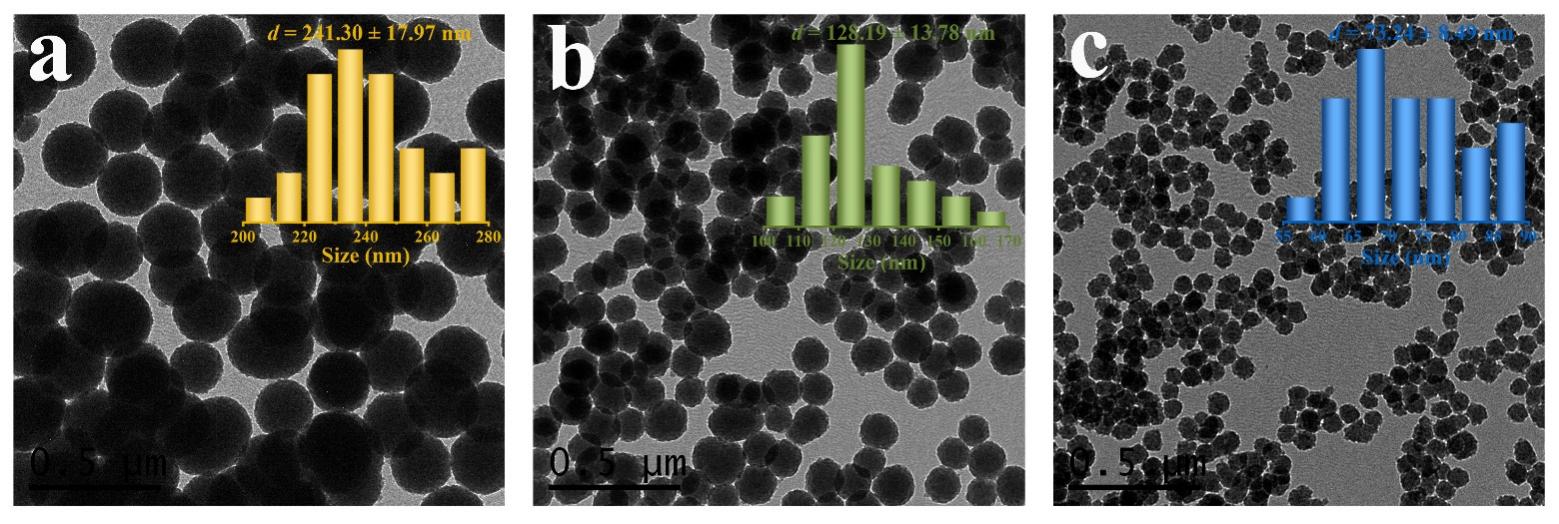


**Figure S1.** The TEM images and size distributions of (a) PDA-1.5, (b) PDA-2.5, and (c) PDA-3.5 nanoparticles.


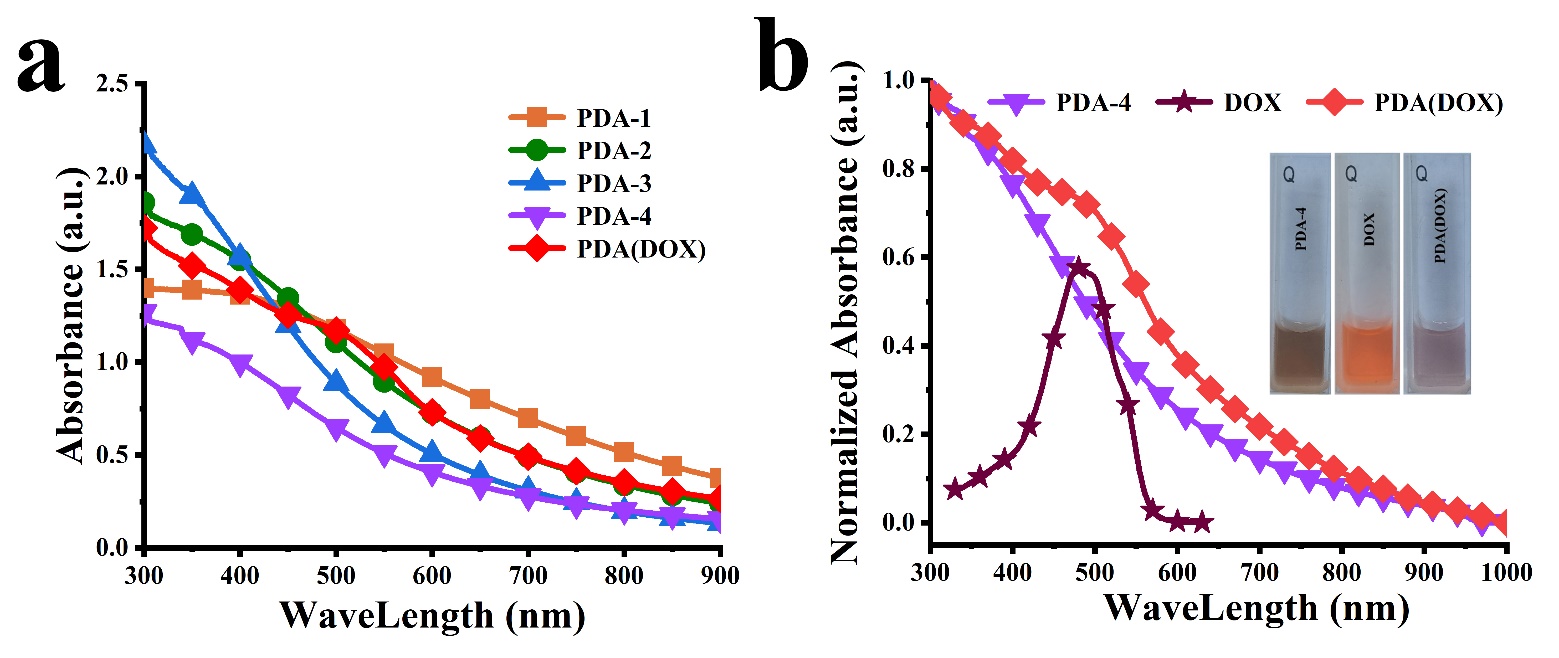


**Figure S2.** (a) The visible-NIR absorbance spectra of PDA-1, PDA-2, PDA-3, PDA-4, and PDA(DOX) with 50 μg/mL PDA concentration. (b) Visible-NIR absorbance spectrum and photos of PDA-4, DOX, and PDA(DOX) dispersion.


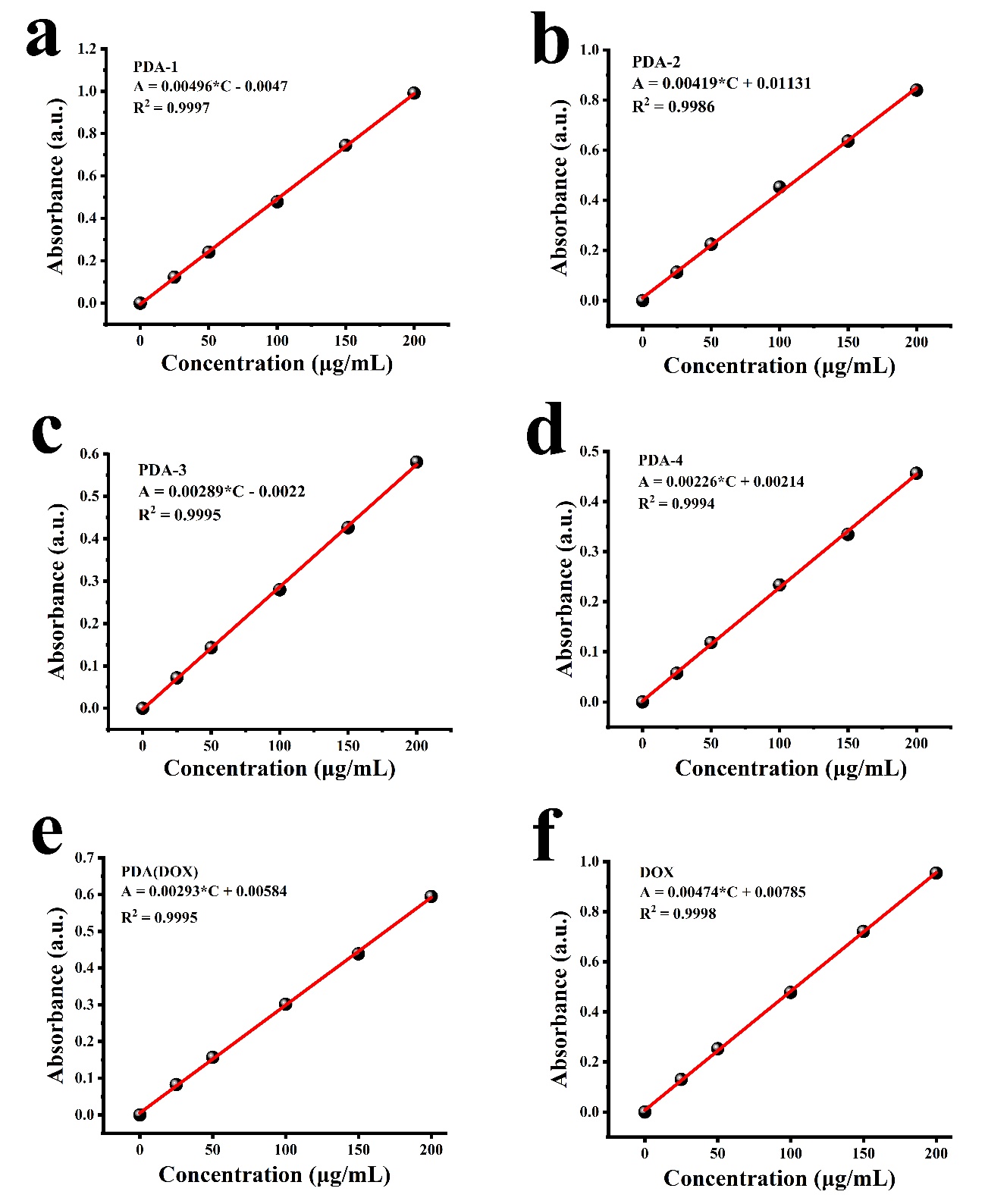


**Figure S3.** The standard curves between the mass concentration of (a) PDA-1; (b) PDA-2; (c) PDA-3; (d) PDA-4; (e) PDA(DOX) nanoparticles dispersion and its absorbance values at 808 nm. (f) The standard curves between the mass concentration of DOX solution and its absorbance values at 488 nm.


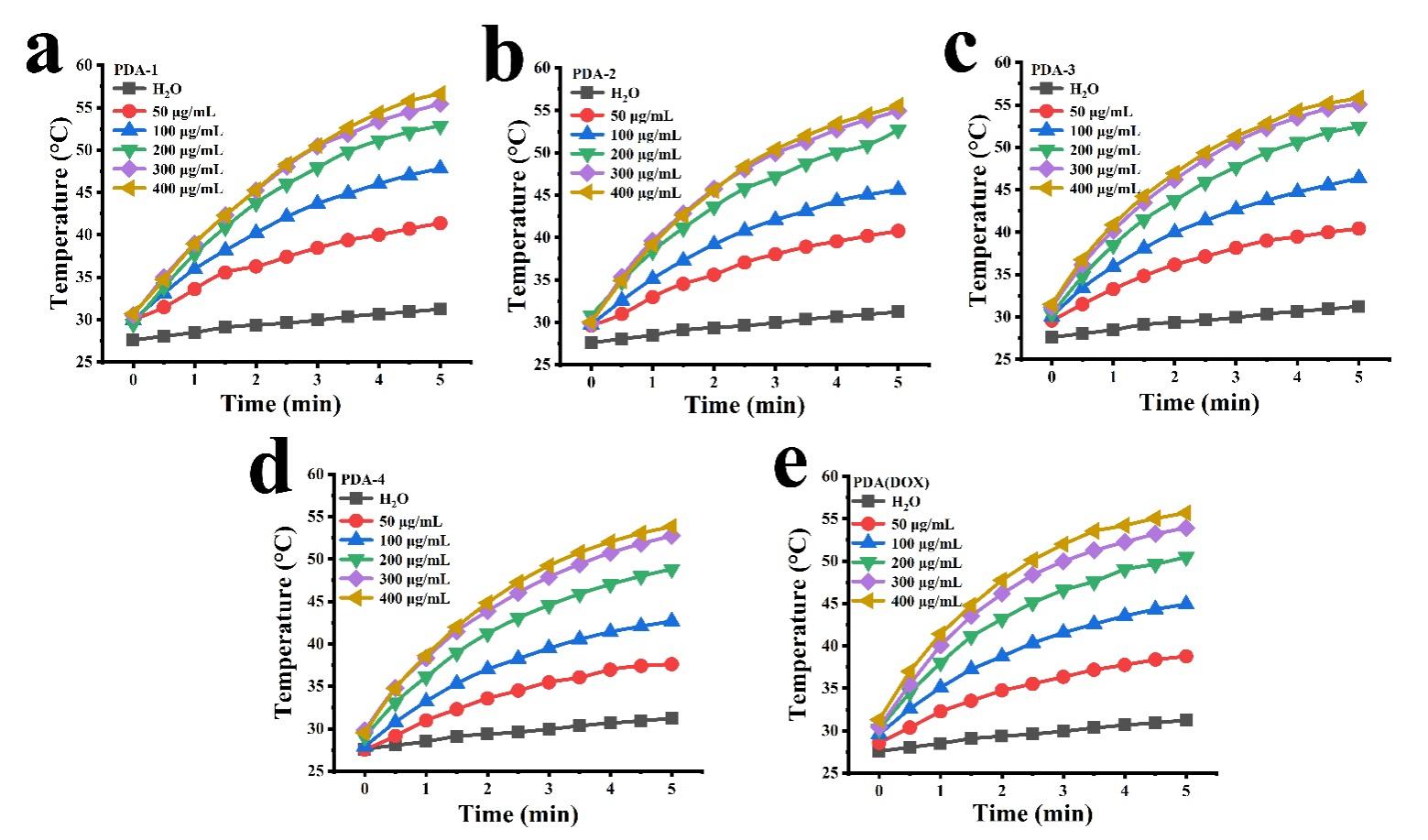


**Figure S4.** The temperature rise curves of (a-d) PDA-*i* and (e) PDA(DOX) dispersions with different PDA concentrations (50, 100, 200, 300, and 400 μg/mL).

**Table S1** The ΔT, I, Q_dis_, τ_s_, *h*s, and A_808 nm_ of PDA and PDA(DOX) nanoparticles for calculating photothermal conversion efficiency η.

| **Samples** | **ΔT=T_max_-T_sur_**  **(℃)** | **I**  **(W)** | **Q_dis_**  **(W)** | **τ_s_**  **(s)** | ***hS***  **(W℃^-1^)** | **A_808 nm_** | **η** |
| --- | --- | --- | --- | --- | --- | --- | --- |
| **PDA-1** | **18.73** | **1.5** | **0.0215** | **421.52** | **0.00996** | **0.636** | **13.68%** |
| **PDA-2** | **14.47** |  |  | **517.19** | **0.00812** | **0.236** | **13.77%** |
| **PDA-3** | **13.06** |  |  | **516.21** | **0.00814** | **0.122** | **20.51%** |
| **PDA-4** | **11.92** |  |  | **556.16** | **0.00755** | **0.083** | **22.65%** |
| **PDA(DOX)** | **14.12** |  |  | **489.01** | **0.00859** | **0.365** | **10.60%** |


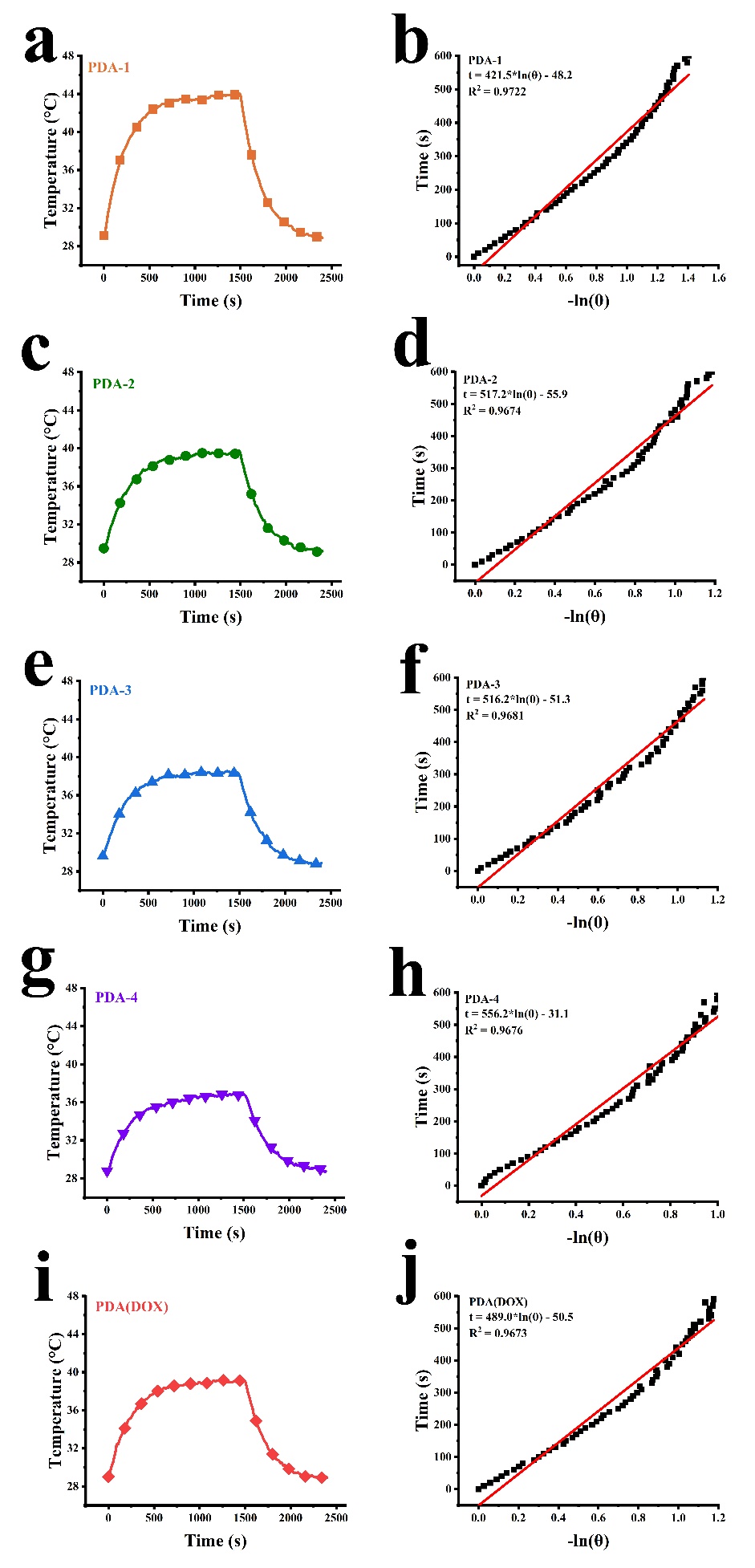


**Figure S5.** Temperature rise and fall curves of (a, c, e, g) PDA-*i* and (i) PDA(DOX) and liner time data versus - lnθ from the cooling of (b, d, f, h) PDA-*i* or (j) PDA(DOX) for obtaining the τ_s_.

**Table S2.** The diameter (D), absorbance value at 808 nm (*A*_808 nm_), volume (*V*_NC_), and mass concentration (C_wt_) of PDA and PDA(DOX) nanoparticles for calculating molar absorption coefficients at 808 nm (ε_808 nm_), absorption cross-section (σ_808 nm_), and absorption efficiency factor (Q_808 nm_).

| **Samples** | **D (nm)** | **A_808 nm_** | ***V*_NC_ (nm^3^)** | **C_wt_ (g L^-1^)** | **σ_808 nm_ (m^2^)** | **Q_808 nm_** | **ε_808 nm_ (M^-1^ cm^-1^)** |
| --- | --- | --- | --- | --- | --- | --- | --- |
| **PDA-1** | **333.73** | **0.636** | **1.95E+07** | **0.05** | **7.10E-14** | **0.81** | **1.86E+11** |
| **PDA-2** | **194.14** | **0.236** | **3.83E+06** | **0.05** | **5.19E-15** | **0.18** | **1.36E+10** |
| **PDA-3** | **101.74** | **0.122** | **5.51E+05** | **0.05** | **3.86E-16** | **0.05** | **1.01E+09** |
| **PDA-4** | **60.3** | **0.083** | **1.15E+05** | **0.05** | **5.47E-17** | **0.02** | **1.43E+08** |
| **PDA(DOX)** | **60.3** | **0.365** | **1.15E+05** | **0.075** | **1.60E-16** | **0.06** | **5.57E+08** |


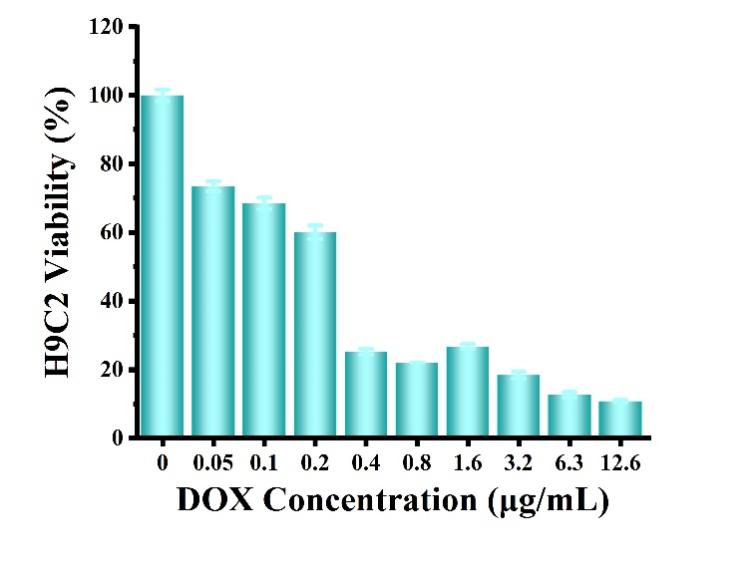


**Figure S6.** The cytotoxicity of various concentrations of DOX on H9C2 cells.


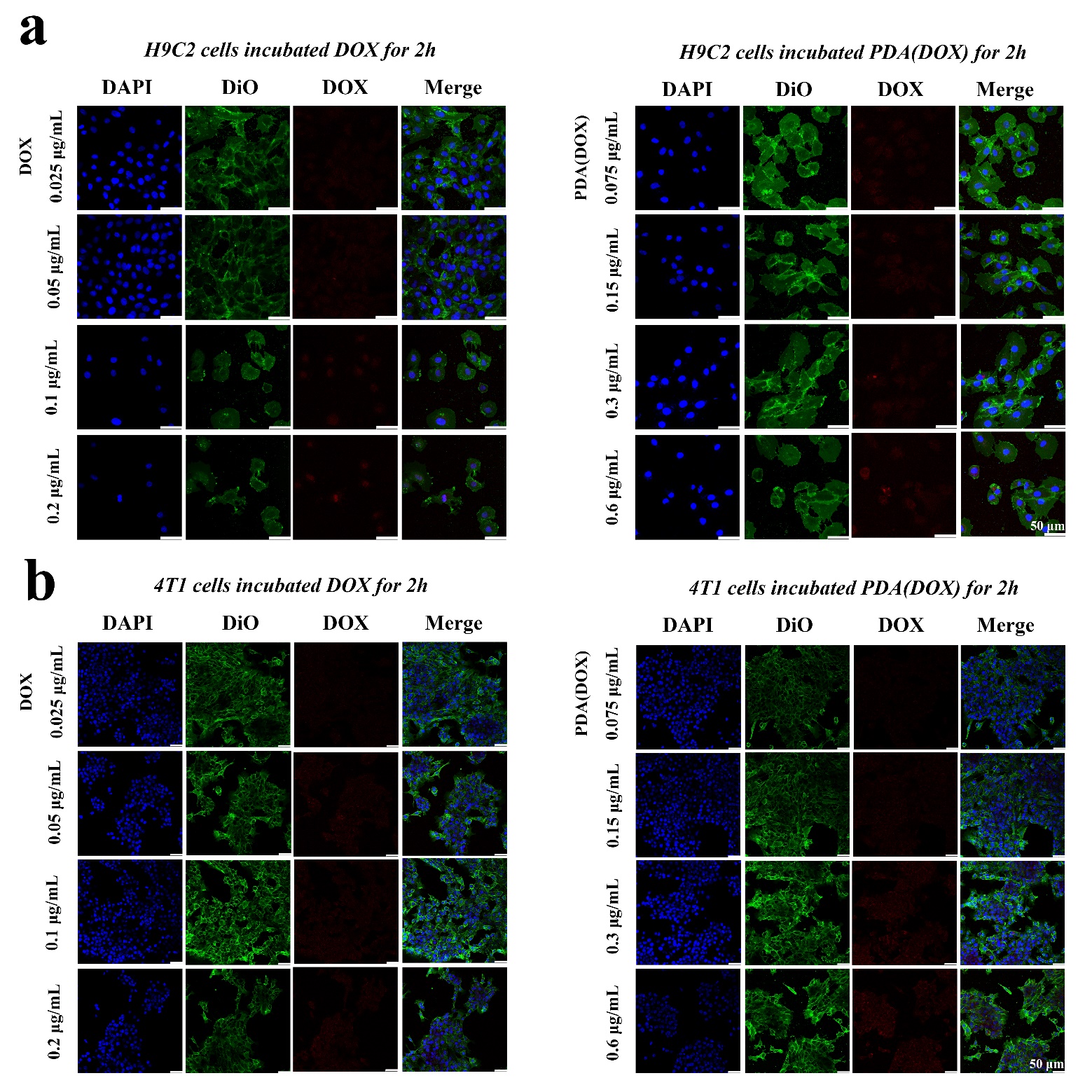


**Figure S7.** Confocal microscope images of H9C2 and 4T1 cells uptake DOX and PDA(DOX) for 2 h with different concentrations.


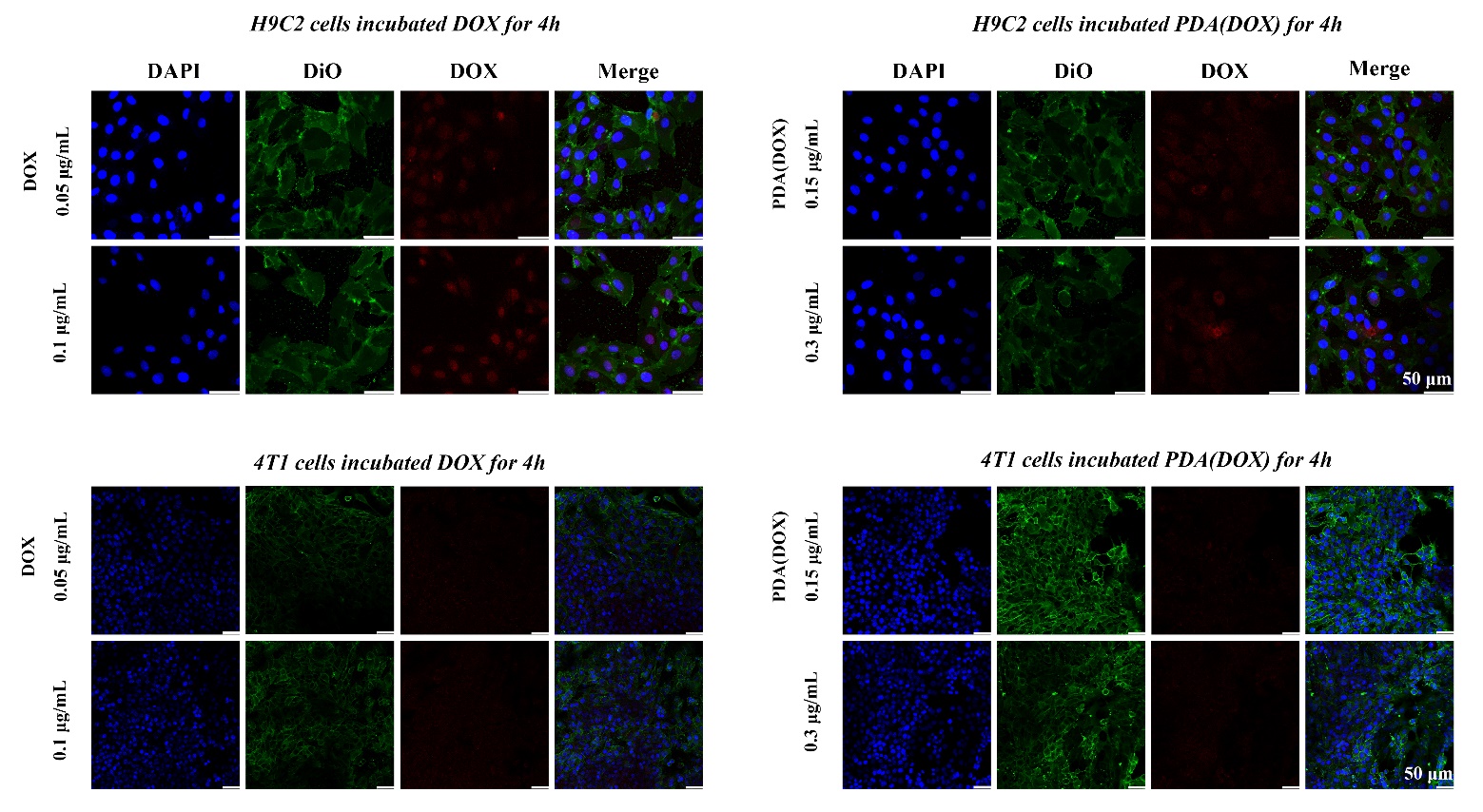


**Figure S8.** Confocal microscope images of H9C2 and 4T1 cells uptake DOX and PDA(DOX) for 4 h with different concentrations.


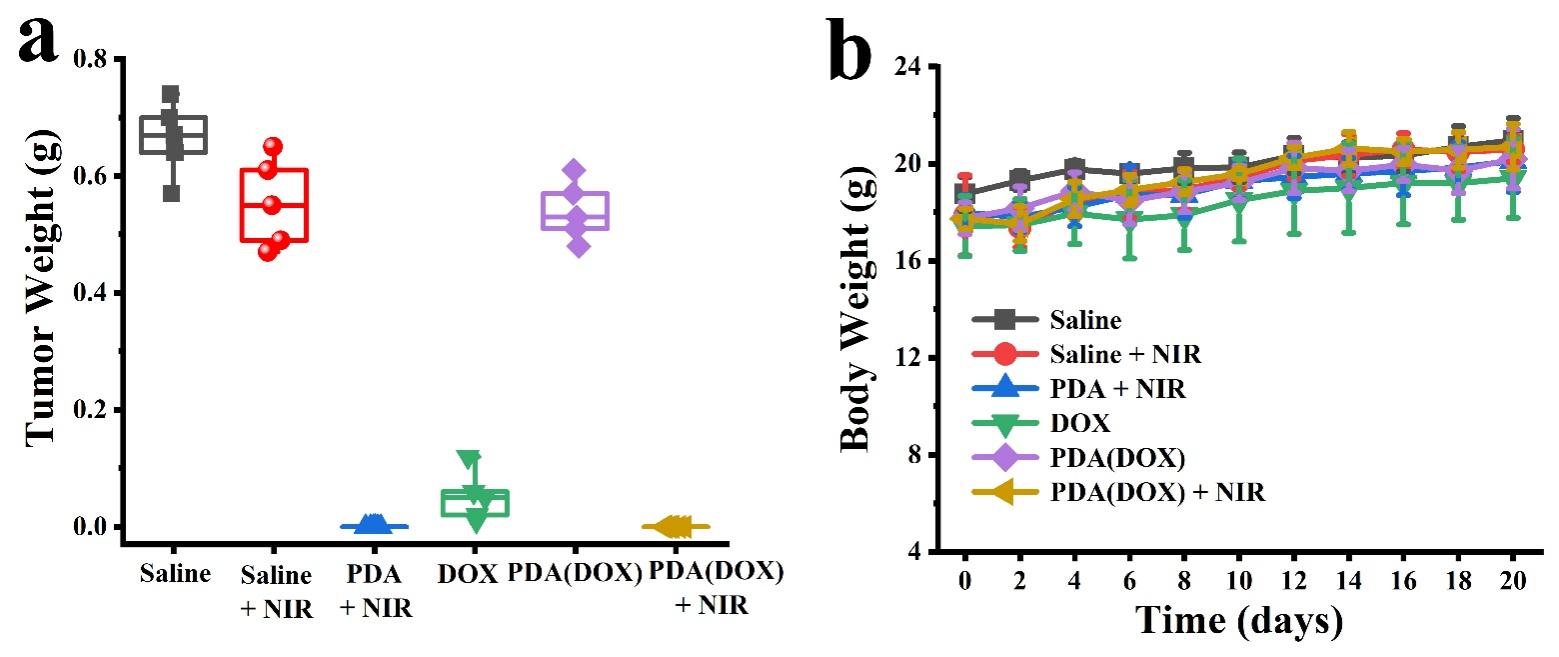


**Figure S9.** (a) Tumor weight of the excised tumors of mice after sacrifice; (b) Body Weights of 4T1 tumor-bearing mice within 21 days after synergetic chemo-photothermal therapy.


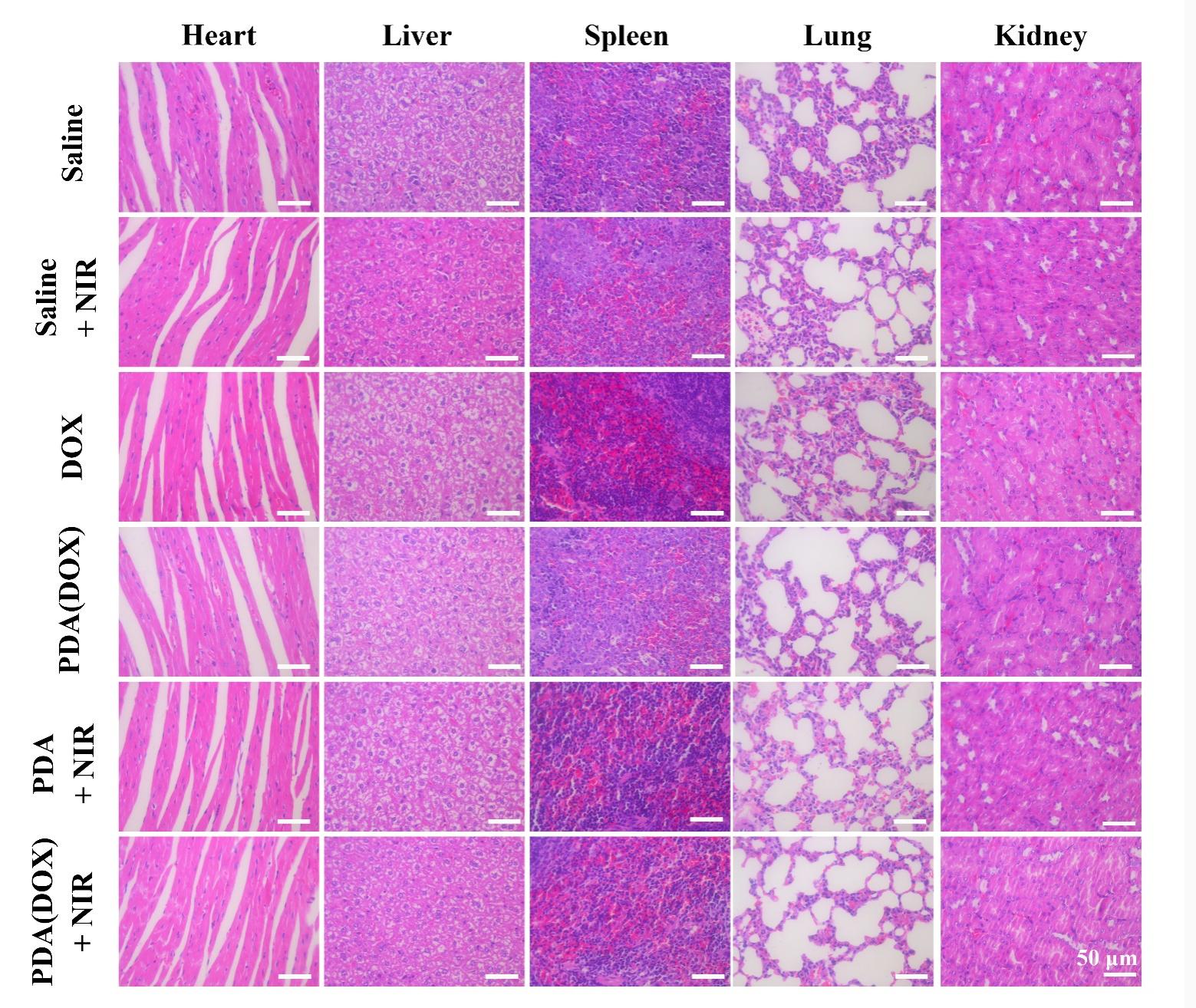


**Figure S10.** Micrographs of H&E-stained major organ slices from mice with synergetic chemo-photothermal therapy were collected after 21 days.


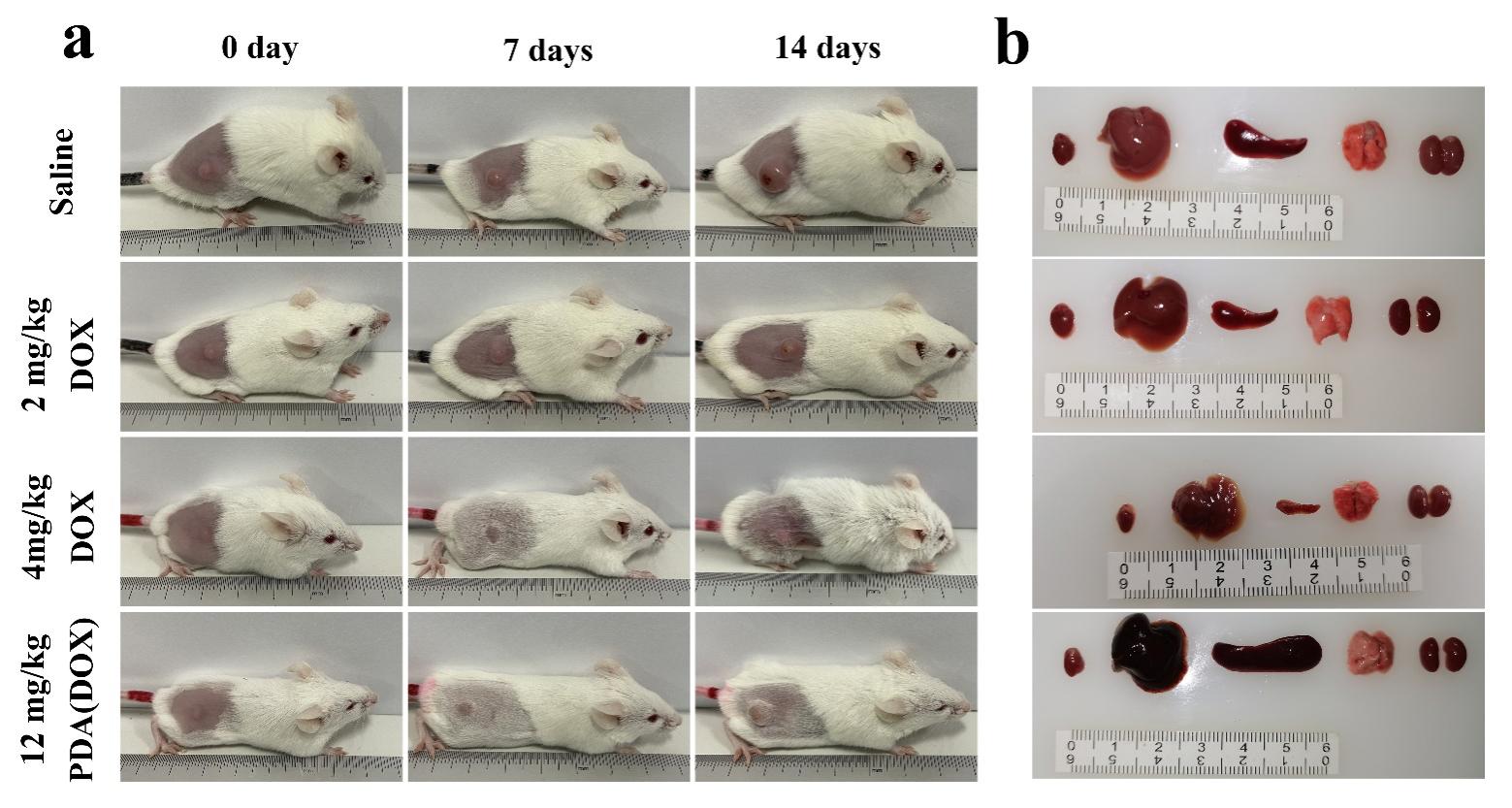


**Figure S11.** (a) 4T1 tumor-bearing mice images on the 0, 7, and 14 days after different treatments and (b) photographs of the major organs of mice after sacrifice.


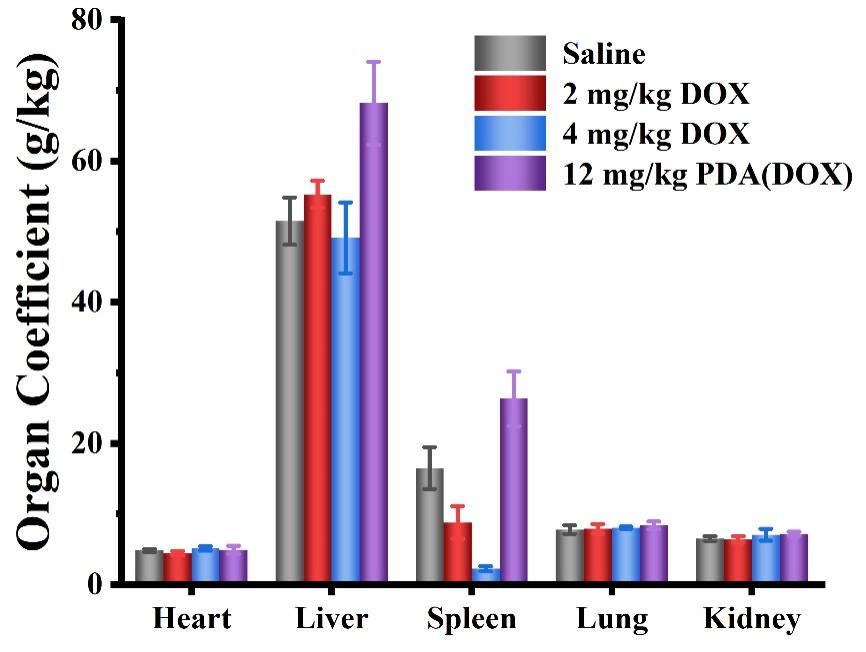


**Figure S12.** Organ Coefficient of chemotherapy mice after sacrifice within 21 days after different treatments.


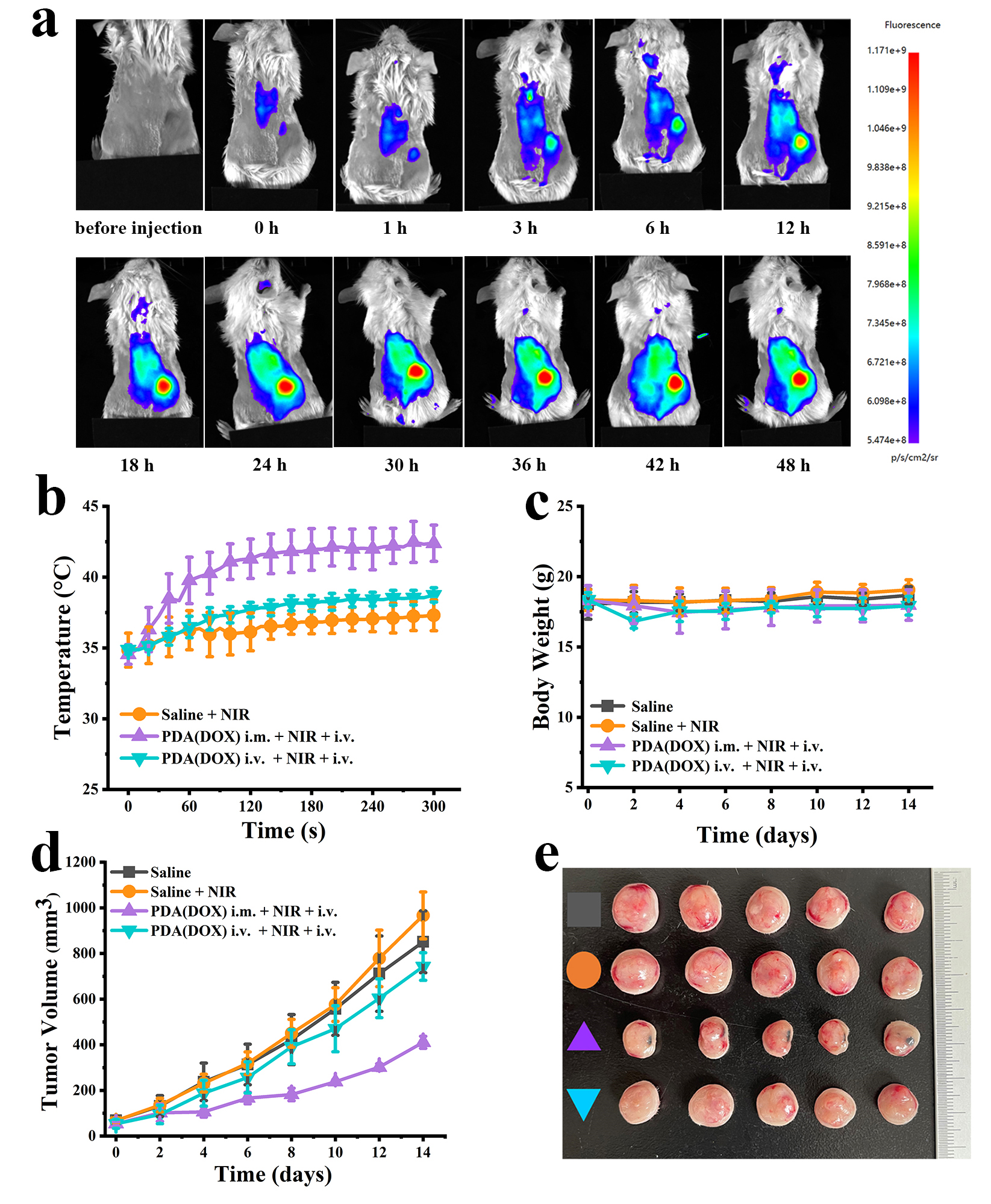


**Figure S13.** *In vivo* synergetic chemo-photothermal therapy of tumor. (a) Fluorescence images of ICG labeled PDA(DOX) nanoparticles at different time points after intravenous injection. (b-d) Temperature rise curves, body weights, and tumor volumes of 4T1 tumor-bearing mice irradiated by 808 nm laser at a laser power of 0.8 W/cm^2^ for 5 min, and then implement chemotherapy; (e) Photographs of the excised tumors of mice after sacrifice.

**Figure S14.** (a) 4T1 tumor-bearing mice images on the 0, 7, and 14 days after different treatments. (b-e) Tumor growth curves from individual 4T1 tumor-bearing Balb/c mice in different treatment groups (NIR: 0.8 W/cm^2^ for 5 min).


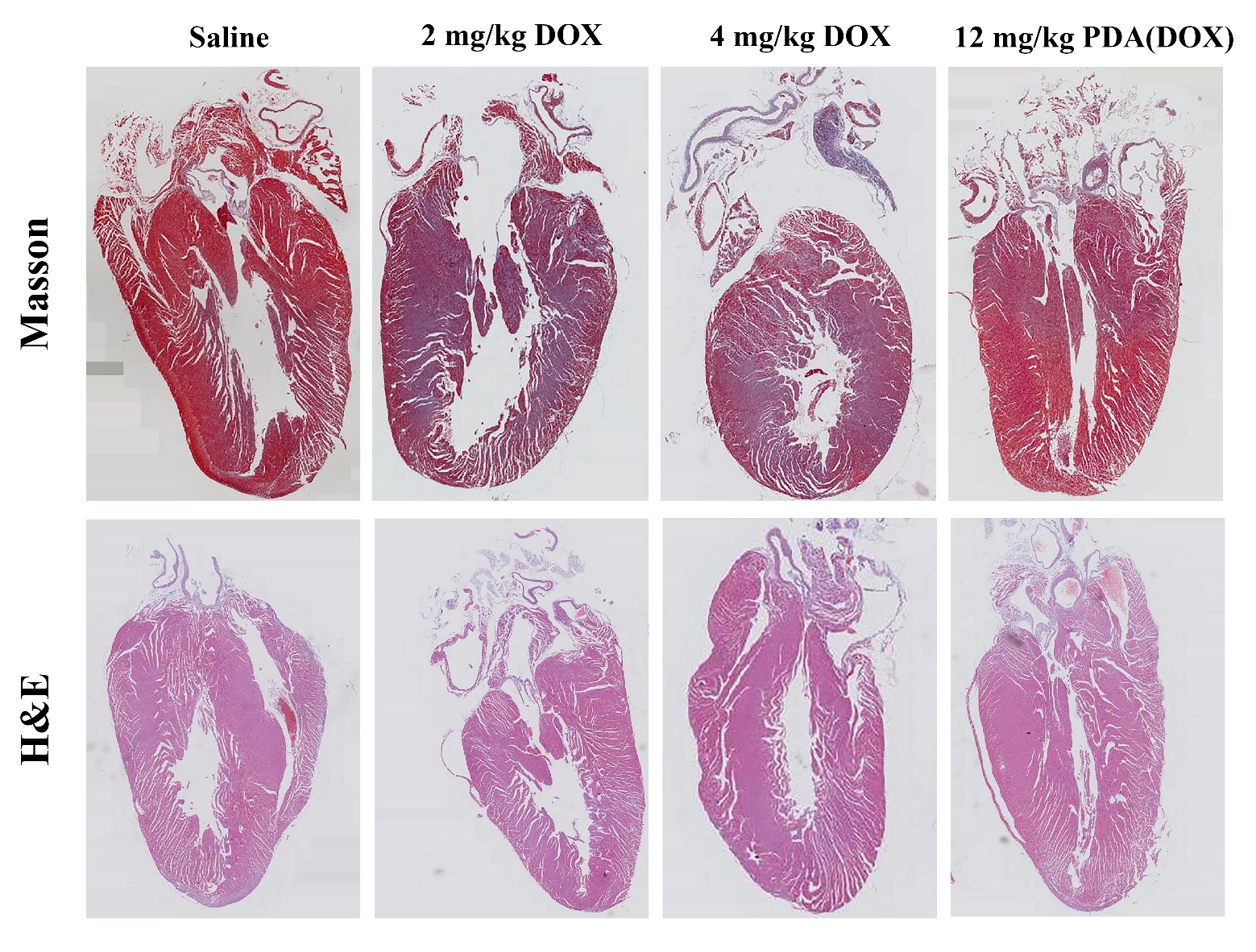


**Figure S15.** H&E and Masson-staining micrographs of major organ slices from different chemotherapy groups after sacrifice within 21 days.
